# New Benzimidazole-1,2,4-Triazole Hybrid Compounds: Synthesis, Anticandidal Activity and Cytotoxicity Evaluation

**DOI:** 10.3390/molecules22040507

**Published:** 2017-03-27

**Authors:** Hülya Karaca Gençer, Ulviye Acar Çevik, Serkan Levent, Begüm Nurpelin Sağlık, Büşra Korkut, Yusuf Özkay, Sinem Ilgın, Yusuf Öztürk

**Affiliations:** 1 Department of Pharmaceutical Microbiology, Faculty of Pharmacy, Anadolu University, Eskişehir 26470, Turkey; 2 Department of Pharmaceutical Chemistry, Faculty of Pharmacy, Anadolu University, Eskişehir 26470, Turkey; uacar@anadolu.edu.tr (U.A.Ç.); serkanlevent@anadolu.edu.tr (S.L.); bnsaglik@anadolu.edu.tr (B.N.S.); 3 Doping and Narcotic Compounds Analysis Laboratory, Faculty of Pharmacy, Anadolu University, Eskişehir 26470, Turkey; yozturk@anadolu.edu.tr; 4 Department of Pharmaceutical Toxicology, Faculty of Pharmacy, Anadolu University, Eskişehir 26470, Turkey; busrakorkut@anadolu.edu.tr (B.K.); silgin@anadolu.edu.tr (S.I.); 5 Department of Pharmacology, Faculty of Pharmacy, Anadolu University, Eskişehir 26470, Turkey

**Keywords:** 1,2,4-triazole, antifungal activity, ergosterol, cytotoxicity, NIH/3T3

## Abstract

Owing to the growing need for antifungal agents, we synthesized a new series 2-((5-(4-(5-substituted-1*H*-benzimidazol-2-yl)phenyl)-4-substituted-4*H*-1,2,4-triazol-3-yl)thio)-1-(substitutedphenyl)ethan-1-one derivatives, which were tested against *Candida* species. The synthesized compounds were characterized and elucidated by FT-IR, ^1^H-NMR, ^13^C-NMR and HR-MS spectroscopies. The synthesized compounds were screened in vitro anticandidal activity against *Candida* species by broth microdiluation methods. In vitro cytotoxic effects of the final compounds were determined by MTT assay. Microbiological studies revealed that compounds **5m**, **5o**, **5r**, **5t**, **5y**, **5ab**, and **5ad** possess a good antifungal profile. Compounds **5w** was the most active derivative and showed comparable antifungal activity to those of reference drugs ketoconazole and fluconazole. Cytotoxicity evaluation of compounds **5m**, **5o**, **5r**, **5w**, **5y**, **5ab** and **5ad** showed that compounds **5w** and **5ad** were the least cytotoxic agents. Effects of these two compounds against ergosterol biosynthesis were observed by LC-MS-MS method, which is based on quantification of ergosterol level in *C. albicans*. Compounds **5w** and **5d** inhibited ergosterol biosynthesis concentration dependently. A fluorescence microscopy study was performed to visualize effect of compound **5w** against *C. albicans* at cellular level. It was determined that compound **5w** has a membrane damaging effect, which may be related with inhibition of biosynthesis of ergosterol.

## 1. Introduction

The *Candida* species are definitely the most important opportunistic fungal pathogens for individuals [1]. Candidiasis can range from non-life threatening mucocutaneous infections to incursive progressions [2]. In the past two decades, a dramatic rise has been observed in the incidence of fungal infections due to increasing number of immunocompromised hosts [3]. The most common reasons of this challenge contain treatment with broad spectrum antibiotics, use of central venous catheters and implantable prosthetic devices, parenteral nutrition, prolonged intensive care unit stay, hemodialysis, immunosuppression, organ transplantation, HIV infection, neutropenia, and use of glucocorticosteroids, chemotherapeutic agents, and immunomodulators [4,5,6,7,8,9]. As a result, antifungal therapy still seems to be one of the most problematic medication issue.

For the therapeutic management of fungal infections, only four important classes of antifungal agents are available in clinical use. These are azoles such as fluconazole, itraconazole, and ketoconazole; polyene macrolides such as Amphotericin B and nystatin; 5-flucytosine; and echinocandins such as caspofungin and micafungin [10]. Among them, azoles are the most widely used antifungal agents on account of their high therapeutic index, broad spectrum of activity and more favorable safety profile [11]. Azole type antifungal drugs have been divided into two groups: triazoles and imidazoles [12]. The most frequently used triazoles are fluconazole and itraconazole that display a wide spectrum of antifungal activity [13]. Several novel triazole antifungal drugs, such as voriconazole, posaconazole, ravuconazole and albaconazole, are available in the market or are at the late stages of clinical trials [14]. These drugs act by competitive inhibition of the lanosterol 14α-demethylase (CYP51A1), which is the key enzyme in sterol biosynthesis of fungi. Selective inhibition of CYP51A1 would cause depletion of ergosterol, a major component of the fungal cell membrane, and accumulation of lanosterol and other 14-methyl sterols resulting in the growth inhibition of fungal cells [14,15].

Benzimidazole compounds have always been important pharmacophores in studies on new antimicrobial agent development. The reason for this special interest can be explained by the structural similarity between purine and benzimidazole [16,17]. Furthermore, the benzimidazole scaffold has an ability to form hydrogen bonds with biological enzymes and receptors and participates in π–π and hydrophobic interactions, which may be related to its mechanism of action [18,19]. Because of these features, several pharmacological and biochemical research have been performed, and many of them have supported the finding that benzimidazole derivatives are potent against various microorganism strains [20,21,22,23,24,25,26,27,28,29,30,31,32,33]. Benzimidazoles can be classified as one the most important group of fungicides with systemic activity and are well-known for their pronounced ability to control a large number of fungal diseases. Thiabendazole, benomyl, carbendazim, chlorfenazole, cypendazole, debacarb, fuberidazole, mecarbinzid, and rabenzazole, which include the benzimidazole moiety, are the main examples of this fungicide class [34,35].

Looking at the antimicrobial importance of benzimidazole and triazole scaffolds, and regarding our previous studies on benzimidazoles [36,37,38,39,40,41,42], we planned to synthesize a hybrid molecules that involve two different pharmacophores. Thus, in this study, 30 new 2-((5-(4-(5-substituted-*1H*-benzimidazol-2-yl)phenyl)-4-substituted-4*H*-1,2,4-triazol-3-yl)thio)-1-(substitutedphenyl)ethan-1-one derivatives (**5a**–**5ad**) were synthesized and investigated for anticandidal activity.

## 2. Results and Discussion

### 2.1. Chemistry

**Scheme 1:**
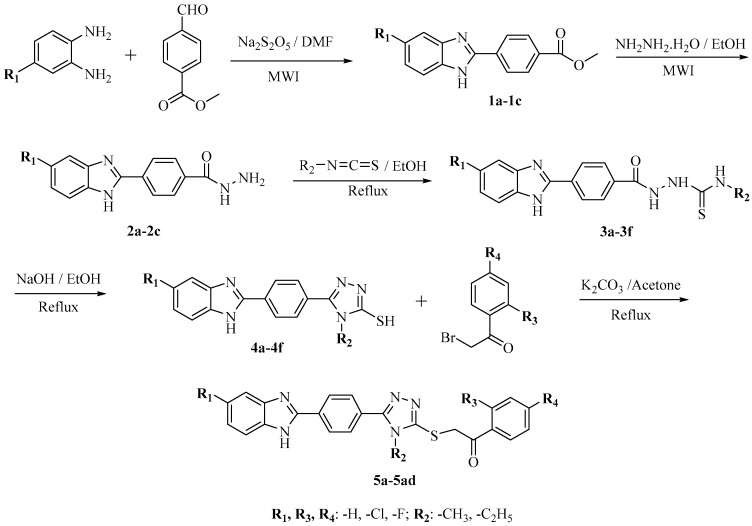
Synthesis route to target compounds **5a**–**5ad**.

The synthesis of target compounds **5a**–**5ad** was performed as outlined in Scheme 1. Initially, methyl 4-(5(6)-substituted-1*H*-benzimidazol-2-yl)benzoate (**1a**–**1c**) derivatives were synthesized by the reaction of methyl 4-formylbenzoate and corresponding o-phenylenediamine in the presence of Na_2_S_2_O_5_. In this step, we applied one of the most used approaches, involving the condensation of *o*-diaminoaromatic compounds with aromatic aldehydes in a two-step procedure, which includes an oxidative cyclodehydrogenation of the corresponding aniline Shiff bases intermediates. In the previous studies, several reagents, such as nitrobenzene [43], 1,4-benzoquinone [44], MnO_2_ [45], benzofuroxan [46], tetracyanoethylene [47], oxone [48], Pb(OAc)_4_ [49], NaHSO_3_ [50], and Na_2_S_2_O_5_ [51] have been used for this purpose. Among these reagents, we preferred Na_2_S_2_O_5_ because it has been reported that high yield reactions could be achieved using Na_2_S_2_O_5_ [52]. In the second reaction step, 4-(5(6)-substituted-1*H*-benzimidazol-2-yl)benzoate (**1a**–**1c**) derivatives were treated with excess of hydrazine hydrate to obtain 4-(5(6)-substituted-1*H*-benzimidazol-2-yl)benzoic acid hydrazides (**2a**–**2c**). In the first and second steps, due to microwave irradiation, the benzimidazole products (**1a**–**1c** and **2a**–**2c**) were obtained in good yields (81%–89%) with a short reaction time (10 min), while in previously reported classical methods [53,54] the similar products were obtained in lower yields with longer reaction times. In the third step, benzimidazole-hydrazides (**2a**–**2c**) were treated with alkylisothiocyanates to afford compounds **3a**–**3f**, which were converted to 5-substituted-1,2,4-triazole-3-thiols (**4a**–**4f**) by the effect of NaOH. In the final step, substitution reaction between 2-bromoacetophenones and compounds **4a**–**4f** gave the target compounds (**5a**–**5ad**). Some characteristics of compounds **5a**–**5ad** are presented in Table 1. The IR, ^1^H-NMR, ^13^C-NMR, and HRMS spectral data (Supplementary materials) were in agreement with the expected structures of compounds **5a**–**5ad**.

Structural elucidation of final compounds was performed by spectral analyses. In the IR spectra, N-H and C=O stretching bands were observed between 3472–3069 cm^−1^ and 1686–1661 cm^−1^, respectively. It is known that benzimidazoles, which contain a hydrogen atom attached to N-1 position readily tautomerize. However, benzimidazoles present a rapid tautomerism which does not allow to observe separate peaks for each tautomers in the NMR spectra [55]. Thus, single signals were assigned for all protons in the NMR analysis. Protons of methylene between the sulfur atom and carbonyl group were recorded as a singlet between 4.58 and 5.03 ppm, and carbon of methylene was observed between 41.08 and 46.21 ppm. Benzimidazole proton at N-1 position gave a singlet between 13.03 and 13.32 ppm. Carbonyl carbons were recorded over 190 ppm as a singlet peak except for difluorophenyl derivatives, which were split into two having 3.0–4.0 Hz coupling constant values. The other hydrogens and carbons were recorded at expected regions. Fluorinated derivatives had coherent carbon-fluorine coupling constants. In the HRMS spectra, all measured mass and isotope ratios were compatible with theoretical values.

### 2.2. Antifungal Activity

Synthesized compounds (**5a**–**5ad**) were evaluated for anticandidal activity against *C. albicans* (ATCC 24433), *C. krusei* (ATCC 6258), *C. parapsilosis* (ATCC 22019) and *C. glabrata* (ATCC 90030). MIC_50_ values were evaluated via fluorometric measurements, using resazurin solution [56,57]. Ketoconazole and fluconazole were used as standard drugs in the activity test. Microdilution test results are presented in Table 2.

Compounds **5a**–**5l**, **5p**, **5q**, **5u**, **5v**, and **5aa** did not show strong activity against *Candida* strains, while stronger anticandidal activity was observed for compounds **5m**–**5o**, **5r**–**5t**, **5w**–**5y**, and **5ab**–**5ad**. *C. albicans* was the most sensitive strain to these compounds. Compound **5w** was the most potent member of the series with an MIC_50_ value of 0.78 µg/mL against all *Candida* species. Compounds **5m**, **5o**, **5r**, **5t**, **5y**, **5ab**, and **5ad** also showed antifungal activity comparable to reference drugs with MIC_50_ values of 0.78–1.56 µg/mL. Compounds **5n**, **5s**, **5v**, and **5ac** displayed moderate antifungal activity (MIC_50_ values = 1.56–3.12 µg/mL).

**Figure 1:**
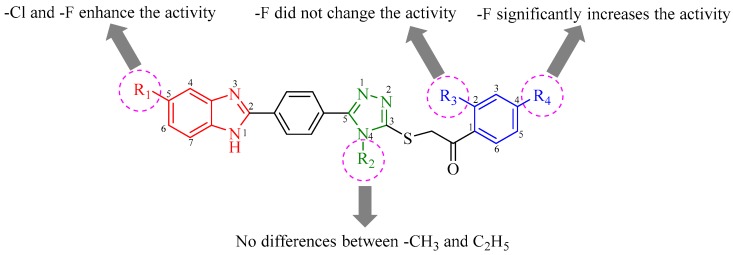
SARs outline of the compounds (**5a**–**5ad**).

The differences in the chemical structures and the antibacterial activity profiles of compounds directed us to discuss structure activity relationships (SARs) (Figure 1). The structural variations of compounds can be classified in three regions. The first one is benzimidazole ring, carrying chloro or fluoro substituents at C-5 position or it is not substituted. The second region is triazole ring in which there is a methyl or ethyl substituents at N-4 position. The last region is phenyl ring of 1-phenyl-1-ethan-1-one substructure that carries chloro or fluoro at C-4 position and dichloro or difluoro substituents at C-2 and C-4 positions. Looking at the chemical structure of the compounds (**5m**–**5o**, **5r**–**5t**, **5w**–**5y**, and **5ab**–**5ad**) that showed stronger anticandidal activity, they commonly bear fluoro substituent at C-4 position of phenyl. Hence, it can be declared that C-4 of phenyl is very important position in terms of anticandidal activity. Fluoro substituents at this position significantly enhance the biological activity. However, compounds **5c**, **5e**, **5h**, and **5j**, which also contain fluoro substituent at C-4 position of phenyl, could not indicate strong antifungal activity. The main structural difference between initial and later compounds is the substituents at C-5 position of benzimidazole. At this position, compounds **5m**–**5o**, **5r**–**5t**, **5w**–**5y**, and **5ab**–**5ad** carry fluoro or chloro substituents, while compounds **5c**, **5e**, **5h**, and **5j** do not include any substituents. Thus, it can be suggested that C-5 position of benzimidazole is essential and fluoro or chloro substitution of this position significantly increases the antifungal activity. The methyl or ethyl substituents at N-4 position of triazole did not cause a meaningful difference on biological activity. Similarly, second fluoro substituent at C-2 position of phenyl in compounds **5e**, **5j**, **5o**, **5t**, **5y**, and **5ad** did not alter the antifungal activity, when compared with monofluoro substitution at C-4 position of phenyl in compounds **5c**, **5h**, **5m**, **5r**, **5w**, and **5ab**.

### 2.3. Prediction of ADME Parameters

Regrettably, significant pharmacological activity is not sufficient for a compound to become drug candidate. An appropriate profile of pharmacokinetics and lowest possible toxicity are also very essential for the new drug candidates and they should be assessed as early as possible in the drug development process. In recent years, an important increase in combinatorial chemistry has arisen and hence preliminary information needed on absorption, distribution, metabolism and excretion (ADME) can be easily provided [58]. Owing to importance of pharmacokinetics, in the present study ADME properties of compounds **5m**–**5o**, **5r**–**5t**, **5w**–**5y**, and **5ab**–**5ad**, which showed strong antifungal activity, were calculated by online Molinspiration property program [59]. This program provides the data of Lipinski′s rule, which evaluates the ADME properties of new compounds and is imperative for the optimization of a biologically active compound. The rule highlights that an orally active drug should not have more than one violation [60].

The theoretical calculations of ADME parameters (molecular weight (MW), log P, topological polar surface are (tPSA), number of hydrogen donors (nON) and acceptors (nOHNH) and volume) and DLS are presented in Table 3 along with the violations of Lipinski’s rule. According to these data, compounds **5m**, **5o**, **5r**, **5w**, **5y**, **5ab**, and **5ad** comply with the rule of Lipinski by causing one violation. However, compounds **5n**, **5s**, **5t**, **5x**, and **5ac** possess two violations and thus they may not display an ideal pharmacokinetics. As a result, it can be declared that compounds **5m**, **5o**, **5r**, **5w**, **5y**, **5ab**, and **5ad** may have good pharmacokinetics profile, which improves their biological importance.

### 2.4. Cytotoxicity Evaluation

Toxicity is a key cause of failure at all steps of the new drug development process. The most part of safety-related problems occur at preclinical phases to predict preclinical safety liabilities earlier in the new drug development process. This approach allows to design and/or selection of better drug candidates that have more potentials to be marketed drugs [61]. Therefore, the MTT cell viability assay, which is suggested for cytotoxicity screening of drug candidates by ISO (10993-5, 2009) was performed [62]. Cytotoxicities of selected compounds (**5m**, **5o**, **5r**, **5w**, **5y**, **5ab**, and **5ad**), displaying strong anticandidal activity and good predicted pharmacokinetics, were determined against NIH/3T3 mouse embryonic fibroblast cell lines (ATCC CRL1658).

The cytotoxicity results of the tested compounds are presented in Table 4. IC_50_ of compounds **5w** (65.28 µg/mL) and **5ad** (119.55 μg/mL) against NIH/3T3 was very higher than their MIC_50_ values (0.78–1.56 µg/mL) against *Candida* strains. These findings show that the antifungal activity of the compounds **5w** and **5ad** is not due to general toxicity, but can be ascribed to their selective action against *Candida* species. Thus, cytotoxicity test findings enhanced the importance of compounds **5w** and **5ad** as anticandidal drug candidates.

### 2.5. Inhibition of Ergosterol Biosynthesis

**Figure 2:**
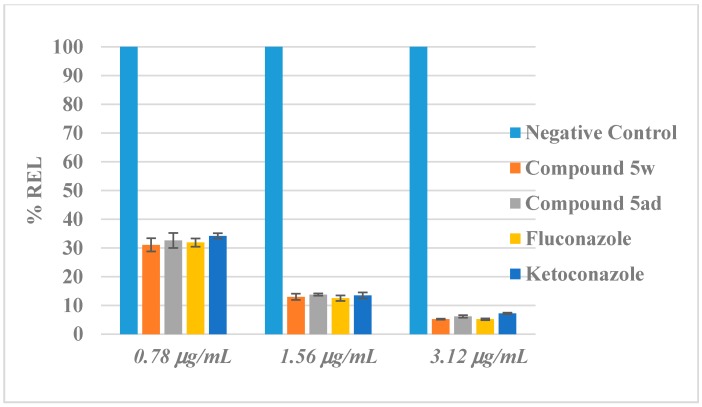
Relative Ergosterol Level (REL) of *C. albicans* (ATCC 24433) after treatment with compounds **5w** and **5ad** fluconazole and ketoconazole.

The most of therapies, planned to treat fungal infections, target in the ergosterol biosynthesis pathway or its end product, ergosterol, a membrane sterol that is unique to fungi. It is the main sterol, and thus is essential for growth and normal membrane function of fungal cell. Besides serving as a bioregulator of membrane fluidity, asymmetry and integrity, it contributes to proper function of membrane-bound enzymes [63]. Hence, we applied an LC-MS-MS (Shimadzu LCMS 8040, Kyoto, Japan) method for quantitative determination of ergosterol content of *C. albicans*. Total intracellular sterols were extracted as reported by Breivik and Owades [64]. Exhibiting stronger anticandidal activities, lower cytotoxicities and better predicted profiles of pharmacokinetics, compounds **5w** and **5ad**, reference agents fluconazole and ketoconazole were used in the assay at MI 0.78 µg/mL, 1.56 µg/mL, and 3.12 µg/mL concentrations, respectively. Ergosterol standard (Product No. 45480, Sigma-Aldrich, Darmstadt, Germany) was used for quantification of ergosterol in both inhibitor-free (negative control) and inhibitor including samples. Ergosterol quantity in negative control samples was regarded as 100%. All concentrations were analyzed in quadruplicate, and the results were expressed as mean ± standard deviation (SD) (Figure 2).

Results showed that the decline in ergosterol levels after treatment with compounds **5w** and **5ad** was noticeable compared to the reference agents. Compounds **5w**, **5ad** and reference agents significantly decreased the level of ergosterol at all tested concentrations. Thus, it can be clearly demonstrated that compounds **5w** and **5ad** have a role in ergosterol biosynthesis pathway.

### 2.6. Fluorescence Microscopy

**Figure 3:**
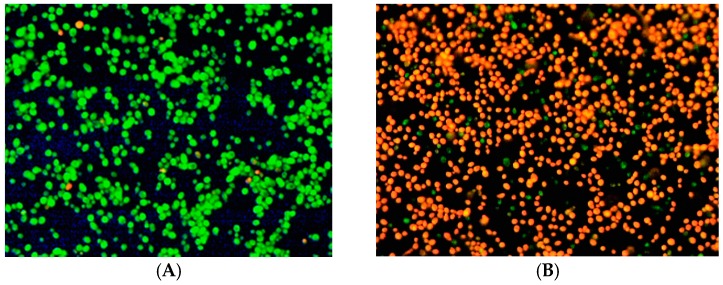
Visualization of *C. albicans* culture under fluorescence microscope: (**A**) *C. albicans*, incubated without compound; and (**B**) *C. albicans*, incubated with compound **5w**.

Ergosterol is the major fungal membrane sterol that regulates membrane fluidity, plasma membrane biogenesis and functions [65]. Hence, the inhibition of ergosterol biosynthesis causes a damage in cell membrane of fungi. To visualize effect of compound **5w**, which possesses the strong anticandidal activity, a low cytotoxicity and a good predicted pharmacokinetics profile, against *C. albicans* at cellular level, a fluorescence microscopy study was performed. Figure 3 shows the cells treated with **5w** and not treated with a compound. Green cells in the photomicrograph represent untreated cells, whereas orange cells depict treated ones with 3.12 µg/mL (4 × MIC_50_) concentration of **5w**. It is known that propidium iodide (PI) enters only cells with damaged membranes, while SYTO-9 marks cells with both damaged and unaffected membranes [66]. Thus, Figure 3 reveals membrane damaging effect of **5w** due to penetration of PI through cell membrane. Consequently, fluorescence microscopic experiments supported the findings observed in inhibition of biosynthesis of ergosterol, which is a vital sterol regulating membrane functions.

## 3. Materials and Methods

### 3.1. Chemistry

Entire chemicals used in the syntheses were purchased from Sigma-Aldrich Chemicals (Sigma-Aldrich Corp., St. Louis, MO, USA) or Merck Chemicals (Merck KGaA, Darmstadt, Germany). Melting points of the synthesized compounds were determined by MP90 digital melting point apparatus (Mettler Toledo, OH, USA) and were uncorrected. ^1^H-NMR and ^13^C-NMR spectra were recorded by a Bruker 300 MHz and 75 MHz digital FT-NMR spectrometer (Bruker Bioscience, Billerica, MA, USA) in DMSO-*d*_6_, respectively. In the NMR spectra splitting patterns were designated as follows: s: singlet; d: doublet; t: triplet; m: multiplet. Coupling constants (*J*) were reported as Hertz. The IR spectra were obtained on a Shimadzu, IR Prestige-21 (Shimadzu, Tokyo, Japan). LC-MS-MS studies were performed on a Shimadzu, 8040 LC-MS-MS spectrophotometer (Shimadzu, Tokyo, Japan). The purities of compounds were checked by TLC on silica gel 60 F254 (Merck KGaA, Darmstadt, Germany).

#### 3.1.1. Synthesis of Methyl 4-(5(6)-substituted-1*H*-benzimidazol-2-yl)benzoate (**1a**–**1c**)

Methyl-4-formyl benzoate (4.8 g, 0.030 mol), sodium disulfite (5.70 g, 0.030 mol) and DMF (5 mL) were added into a vial (30 mL) of microwave synthesis reactor (Anton-Paar, Monowave 300, Graz, Austria). The reaction mixture, was heated under conditions of 240 °C and 10 bar for 5 min. The mixture was cooled down, 5-substituted-1,2-phenylenediamine (0.028 mol) was added and then reaction mixture was kept under the same reaction conditions in microwave reactor. After cooling, the mixture was poured into iced-water, precipitated product was washed with water, dried and recrystallized from ethanol [53,54].

#### 3.1.2. Synthesis of 4-(5(6)-Substituted-1*H*-benzimidazol-2-yl)benzoic Acid Hydrazide (**2a**–**2c**)

Methyl 4-(5(6)-substituted-1*H*-benzimidazol-2-yl)benzoate (**1a**–**1c**) (0.025 mol) in ethanol (15 mL) and excess of hydrazine hydrate (5 mL) were put into a vial (30 mL) of microwave synthesis reactor (Anton-Paar Monowave 300, Graz, Austria). The reaction mixture was kept under the conditions of 150 °C and 10 bar for 10 min. After cooling, the mixture was poured into iced-water, precipitated product was washed with water, dried and recrystallized from ethanol [53,54].

#### 3.1.3. Synthesis of 2-(4-(5(6)-Substituted-1*H*-benzimidazol-2-yl)benzoyl)-*N*’-methyl/ethyl-hydrazine-1-carbothioamide (**3a**–**3f**)

4-(5(6)-Substituted-1*H*-benzimidazol-2-yl)benzoic acid hydrazide (**2a**–**2c**) (0.020 mol) and corresponding alkylisothiocyanate (0.024 mol) was dissolved in ethanol. The reaction mixture was refluxed for 2 h. The precipitated product is filtered, washed with ethanol and dried.

*2-(4-(1H-Benzimidazol-2-yl)-N-methylhydrazine-1-carbothioamide* (**3a**): FTIR (ATR, cm^−1^): 3268, 3154 (N-H), 1682 (C=O), 1215 (C=S), 1106, 819, 735. ^1^H-NMR (300 MHz, DMSO-*d*_6_): δ = 2.85 (3H, s, -CH_3_), 7.19–7.28 (2H, m, Benzimidazole C-H), 7.57 (1H, d, *J =* 7.20 Hz, Benzimidazole C-H), 7.70 (1H, d, *J =* 7.20 Hz Benzimidazole C-H), 7.90 (2H, d, *J =* 8.40 Hz, Ar. C-H), 8.34 (2H, d, *J =* 8.40 Hz, Ar. C-H), 9.23 (1H, s, N-H), 10.04 (1H, s, N-H), 10.24 (1H, s, N-H), 12.87 (1H, s, Benzimidazole N-H). ^13^C-NMR (75 MHz, DMSO-*d_6_*): δ = 31.52, 119.54, 122.43, 127.29, 129.66, 130.47, 131.99, 134.46, 139.02, 143.84, 151.21, 167.87, 182.21. HRMS (*m*/*z*): [M + H]^+^ calcd. for C_16_H_15_N_5_OS: 322.1076; found: 322.1069.

*2-(4-(1H-Benzimidazol-2-yl)-N-ethylhydrazine-1-carbothioamide* (**3b**): FTIR (ATR, cm^−1^): 3252, 3192 (N-H), 1678 (C=O), 1219 (C=S), 1100, 811, 733. ^1^H-NMR (300 MHz, DMSO-*d*_6_): δ = 1.21 (3H, t, *J* = 7.20 Hz, -CH_3_), 4.06 (2H, q, *J* = 7.20 Hz, -CH_2_), 7.20–7.27 (2H, m, Benzimidazole C-H), 7.56 (1H, d, *J =* 7.20 Hz, Benzimidazole C-H), 7.71 (1H, d, *J =* 7.20 Hz Benzimidazole C-H), 7.92 (2H, d, *J =* 8.40 Hz, Ar. C-H), 8.33 (2H, d, *J =* 8.40 Hz, Ar. C-H), 9.35 (1H, s, N-H), 10.03 (1H, s, N-H), 10.34 (1H, s, N-H), 12.96 (1H, s, Benzimidazole N-H). ^13^C-NMR (75 MHz, DMSO-*d*_6_): δ = 16.32, 32.34, 119.66, 123.12, 127.21, 129.66, 130.22, 132.45, 135.19, 139.02, 144.46, 152.09, 167.32, 181.11. HRMS (*m*/*z*): [M + H]^+^ calcd. for C_17_H_17_N_5_OS: 340.1232; found: 340.1234.

*2-(4-(6-Chloro-1H-benzimidazol-2-yl)-N-methylhydrazine-1-carbothioamide* (**3c**): FTIR (ATR, cm^−1^): 3288, 3143 (N-H), 1677 (C=O), 1208 (C=S), 1189, 844, 736. ^1^H-NMR (300 MHz, DMSO-*d*_6_): δ = 2.88 (3H, s, -CH_3_), 7.27 (1H, d, *J =* 8.70 Hz, Benzimidazole C-H), 7.59 (1H, s, Benzimidazole C-H), 7.75 (1H, d, *J =* 8.70 Hz, Benzimidazole C-H), 7.90 (2H, d, *J =* 8.40 Hz, Ar. C-H), 8.36 (2H, d, *J =* 8.40 Hz, Ar. C-H), 9.28 (1H, s, N-H), 10.03 (1H, s, N-H), 10.26 (1H, s, N-H), 13.24 (1H, s, Benzimidazole N-H). ^13^C-NMR (75 MHz, DMSO-*d*_6_): δ = 31.11, 114.36, 117.17, 122.78, 123.47, 127.42, 129.68, 132.25, 134.27, 136.93 142.19, 153.52, 166.25, 184.44. HRMS (*m*/*z*): [M + H]^+^ calcd. for C_16_H_14_N_5_ClOS: 360.0686; found: 360.0694.

*2-(4-(6-Chloro-1H-benzimidazol-2-yl)-N-ethylhydrazine-1-carbothioamide* (**3d**): FTIR (ATR, cm^−1^): 3295, 3143 (N-H), 1677 (C=O), 1222 (C=S), 1145, 849, 735. ^1^H-NMR (300 MHz, DMSO-*d*_6_): δ = 1.21 (3H, t, *J =* 7.15 Hz, -CH_3_), 4.06 (2H, q, *J =* 7.15 Hz, -CH_2_), 7.27 (1H, d, *J =* 8.65 Hz, Benzimidazole C-H), 7.61 (1H, s, Benzimidazole CH), 7.77 (1H, d, *J =* 8.65 Hz, Benzimidazole C-H), 7.93 (2H, d, *J =* 8.40 Hz, Ar. C-H), 8.33 (2H, d, *J =* 8.40 Hz, Ar. C-H), 9.26 (1H, s, N-H), 10.05 (1H, s, N-H), 10.25 (1H, s, N-H), 13.24 (1H, s, Benzimidazole N-H). ^13^C-NMR (75 MHz, DMSO-*d*_6_): δ = 15.98, 33.02, 113.82, 119.01, 122.78, 123.49, 126.21, 130.10, 132.77, 134.27, 136.96, 142.21, 153.52, 167.56, 187.22. HRMS (*m*/*z*): [M + H]^+^ calcd. for C_17_H_16_N_5_ClOS: 374.0842; found: 374.0852.

*2-(4-(6-Fluoro-1H-benzimidazol-2-yl)-N-methylhydrazine-1-carbothioamide* (**3e**): FTIR (ATR, cm^−1^): 3254, 3168 (N-H), 1676 (C=O), 1214 (C=S), 1138, 843, 708. ^1^H-NMR (300 MHz, DMSO-*d*_6_): δ = 2.92 (3H, s, -CH_3_), 7.10–7.16 (1H, m, Benzimidazole C-H), 7.46 (1H, br. s, Benzimidazole C-H), 7.51–7.59 (1H, m, Benzimidazole C-H), 7.88 (2H, d, *J =* 8.45 Hz, Ar. C-H), 8.36 (2H, d, *J =* 8.45 Hz, Ar. C-H), 9.24 (1H, s, N-H), 10.08 (1H, s, N-H), 10.25 (1H, s, N-H), 13.23 (1H, s, Benzimidazole N-H). ^13^C-NMR (75 MHz, DMSO-*d*_6_): δ = 32.10, 99.91 (d, *J =* 26.7 Hz), 105.13 (d, *J =* 23.3 Hz), 112.60 (d, *J =* 10.3 Hz), 127.54 (d, *J =* 9.1 Hz), 128.92, 130.18, 131.52, 135.48, 151.80 (d, *J =* 2.8 Hz), 152.37, 159.09 (d, *J =* 231.4 Hz), 166.93, 191.12. HRMS (*m*/*z*): [M + H]^+^ calcd. for C_17_H_16_N_5_FOS: 358.1138; found: 358.1141.

*2-(4-(6-Fluoro-1H-benzimidazol-2-yl)-N-ethylhydrazine-1-carbothioamide* (**3f**): FTIR (ATR, cm^−1^): 3273, 3182 (N-H), 1672 (C=O), 1206 (C=S), 1141, 843, 713. ^1^H-NMR (300 MHz, DMSO-*d*_6_): δ = 1.24 (3H, t, *J =* 7.10 Hz, -CH_3_), 4.02 (2H, q, *J =* 7.10 Hz, -CH_2_), 7.08–7.16 (1H, m, Benzimidazole C-H), 7.45 (1H, br. s, Benzimidazole C-H), 7.52–7.60 (1H, m, Benzimidazole C-H), 7.85 (2H, d, *J =* 8.40 Hz, Ar. C-H), 8.36 (2H, d, *J =* 8.40 Hz, Ar. C-H), 9.25 (1H, s, N-H), 10.08 (1H, s, N-H), 10.26 (1H, s, N-H), 13.18 (1H, s, Benzimidazole N-H). ^13^C-NMR (75 MHz, DMSO-*d*_6_): δ = 15.99, 32.49, 100.04 (d, *J =* 26.7 Hz), 105.14 (d, *J =* 23.5 Hz), 112.62 (d, *J =* 10.3 Hz), 127.58 (d, *J =* 9.3 Hz), 128.96, 130.20, 131.64, 136.15, 151.81 (d, *J =* 2.4 Hz), 152.45, 159.19 (d, *J =* 233.2 Hz), 167.06, 189.03. HRMS (*m*/*z*): [M + H]^+^ calcd. for C_18_H_18_N_5_FOS: 372.1294; found: 372.1286.

#### 3.1.4. Synthesis of 5-(4-((5(6)-Substituted-1*H*-benzimidazol-2-yl)phenyl)-4-methyl/ethyl-4*H*-1,2,4-triazole-3-thiol (**4a**–**4f**)

NaOH (0.72 g, 0.018 mol) was dissolved in ethanol (50 mL) and 2-(4-(5(6)-substituted-1*H*-benzimidazol-2-yl)benzoyl)-*N*’-methyl/ethyl-hydrazine-1-carbothioamide (**3a**–**3f**) (0.015 mol) was added. The reaction mixture was refluxed for 2 h. After completion of reaction, the mixture is acidified with HCl until pH = 2. The precipitated product is filtered and washed with water, dried and then recrystallized from ethanol.

*5-(4-(1H-Benzimidazol-2-yl)phenyl)-4-methyl-4H-1,2,4-triazole-3-thiol* (**4a**): FTIR (ATR, cm^−1^): 3201 (N-H), 2612 (S-H), 1121, 825, 738. ^1^H-NMR (300 MHz, DMSO-*d*_6_): δ = 3.74 (3H, s, -CH_3_), 7.19–7.28 (2H, m, Benzimidazole C-H), 7.58 (1H, d, *J =* 7.20 Hz, Benzimidazole C-H), 7.72 (1H, d, *J =* 7.20 Hz Benzimidazole C-H), 7.98 (2H, d, *J =* 8.50 Hz, Ar. C-H), 8.37 (2H, d, *J =* 8.50 Hz, Ar. C-H), 12.87 (1H, s, Benzimidazole N-H), 13.08 (1H, s, SH). ^13^C-NMR (75 MHz, DMSO-*d*_6_): δ = 43.86, 118.62, 123.84, 127.83, 129.94, 132.15, 134.82, 141.43, 151.21, 154.11, 163.42. HRMS (*m*/*z*): [M + H]^+^ calcd. for C_16_H_13_N_5_S: 308.0970; found: 308.0979.

*5-(4-(1H-Benzimidazol-2-yl)phenyl)-4-ethyl-4H-1,2,4-triazole-3-thiol* (**4b**): FTIR (ATR, cm^−1^): 3232 (N-H), 2618 (S-H), 1100, 821, 738. ^1^H-NMR (300 MHz, DMSO-*d*_6_): δ = 1.28 (3H, t, *J =* 7.20 Hz, -CH_3_), 4.11 (2H, q, *J =* 7.20 Hz, -CH_2_), 7.19–7.26 (2H, m, Benzimidazole C-H), 7.60 (1H, d, *J =* 7.20 Hz, Benzimidazole C-H), 7.74 (1H, d, *J =* 7.20 Hz Benzimidazole C-H), 7.97 (2H, d, *J =* 8.50 Hz, Ar. C-H), 8.33 (2H, d, *J =* 8.40 Hz, Ar. C-H), 12.96 (1H, s, Benzimidazole N-H), 13.06 (1H, s, SH). ^13^C-NMR (75 MHz, DMSO-*d*_6_): δ = 16.87, 44.54, 119.05, 123.89, 127.65, 130.12, 132.18, 135.26, 142.33, 150.96, 153.86, 163.12. HRMS (*m*/*z*): [M + H]^+^ calcd. for C_17_H_15_N_5_S: 322.1126; found: 322.1129.

*5-(4-(6-Chloro-1H-benzimidazol-2-yl)phenyl)-4-methyl-4H-1,2,4-triazole-3-thiol* (**4c**): FTIR (ATR, cm^−1^): 3214 (N-H), 2634 (S-H), 1105, 817, 740. ^1^H-NMR (300 MHz, DMSO-*d*_6_): δ = 3.72 (3H, s, -CH_3_), 7.29 (1H, d, *J =* 8.70 Hz, Benzimidazole C-H), 7.60 (1H, s, Benzimidazole C-H), 7.72 (1H, d, *J =* 8.70 Hz, Benzimidazole C-H), 7.96 (2H, d, *J =* 8.40 Hz, Ar. C-H), 8.39 (2H, d, *J =* 8.40 Hz, Ar. C-H), 13.08 (1H, s, SH), 13.24 (1H, s, Benzimidazole N-H). ^13^C-NMR (75 MHz, DMSO-*d*_6_): δ = 43.18, 114.38, 118.21, 122.65, 123.74, 126.99, 130.61, 132.28, 134.27, 136.36, 142.16, 150.63, 153.48, 165.44. HRMS (*m*/*z*): [M + H]^+^ calcd. for C_16_H_12_N_5_ClS: 342.0580; found: 342.0581.

*5-(4-(6-Chloro-1H-benzimidazol-2-yl)phenyl)-4-ethyl-4H-1,2,4-triazole-3-thiol* (**4d**): FTIR (ATR, cm^−1^): 3210 (N-H), 2635 (S-H), 1120, 820, 736. ^1^H-NMR (300 MHz, DMSO-*d*_6_): δ = 1.28 (3H, t, *J =* 7.20 Hz, -CH_3_), 4.11 (2H, q, *J =* 7.20 Hz, -CH_2_), 7.31 (1H, d, *J =* 8.65 Hz, Benzimidazole C-H), 7.60 (1H, s, Benzimidazole C-H), 7.75 (1H, d, *J =* 8.65 Hz, Benzimidazole C-H), 7.96 (2H, d, *J =* 8.40 Hz, Ar. -CH), 8.42 (2H, d, *J =* 8.40 Hz, Ar. C-H), 13.07 (1H, s, SH), 13.21 (1H, s, Benzimidazole N-H). ^13^C-NMR (75 MHz, DMSO-*d*_6_): δ = 16.12, 45.26, 115.39, 119.13, 122.65, 125.01, 128.10, 130.16, 132.29, 134.27, 137.22, 143.01, 151.43, 154.23, 166.47. HRMS (*m*/*z*): [M + H]^+^ calcd. for C_17_H_14_N_5_ClS: 356.0737; found: 356.0742.

*5-(4-(6-Fluoro-1H-benzimidazol-2-yl)phenyl)-4-methyl-4H-1,2,4-triazole-3-thiol* (**4e**): FTIR (ATR, cm^−1^): 3199 (N-H), 2647 (S-H), 1138, 843, 708. ^1^H-NMR (300 MHz, DMSO-*d*_6_): δ = 3.74 (3H, s, -CH_3_), 7.10–7.18 (1H, m, Benzimidazole C-H), 7.48 (1H, br. s, Benzimidazole C-H), 7.51–7.60 (1H, m, Benzimidazole C-H), 7.89 (2H, d, *J =* 8.50 Hz, Ar. C-H), 8.37 (2H, d, *J =* 8.50 Hz, Ar. C-H), 13.06 (1H, s, S-H), 13.25 (1H, s, Benzimidazole N-H). ^13^C-NMR (75 MHz, DMSO-*d*_6_): δ = 32.21, 99.46 (d, *J =* 26.7 Hz), 105.21 (d, *J =* 23.6 Hz), 112.61 (d, *J =* 10.3 Hz), 127.52 (d, *J =* 9.4 Hz), 128.96, 130.18, 132.16, 135.48, 151.71 (d, *J =* 2.8 Hz), 152.21, 154.23, 159.09 (d, *J =* 231.4 Hz), 167.12. HRMS (*m*/*z*): [M + H]^+^ calcd. for C_16_H_12_N_5_FS: 326.0876; found: 326.0886.

*5-(4-(6-Fluoro-1H-benzimidazol-2-yl)phenyl)-4-ethyl-4H-1,2,4-triazole-3-thiol* (**4f**): FTIR (ATR, cm^−1^): 3210 (N-H), 2651 (S-H),), 1121, 846, 716. ^1^H-NMR (300 MHz, DMSO-*d*_6_): δ = 1.28 (3H, t, *J =* 7.20 Hz, -CH_3_), 4.11 (2H, q, *J =* 7.20 Hz, -CH_2_), 7.12–7.19 (1H, m, Benzimidazole C-H), 7.49 (1H, br. s, Benzimidazole C-H), 7.52–7.61 (1H, m, Benzimidazole C-H), 7.91 (2H, d, *J =* 8.40 Hz, Ar. C-H), 8.40 (2H, d, *J =* 8.40 Hz, Ar. C-H), 13.07 (1H, s, S-H), 13.20 (1H, s, Benzimidazole N-H). ^13^C-NMR (75 MHz, DMSO-*d*_6_): δ = 16.12, 45.26, 99.63 (d, *J =* 26.2 Hz), 105.20 (d, *J =* 23.5 Hz), 112.62 (d, *J =* 10.4 Hz), 127.52 (d, *J =* 9.4 Hz), 128.89, 130.21, 132.18, 136.12, 151.74 (d, *J =* 2.9 Hz), 151.96, 153.96, 159.16 (d, *J =* 232.8 Hz), 166.41. HRMS (*m*/*z*): [M + H]^+^ calcd. for C_17_H_14_N_5_FS: 340.1032; found: 340.1041.

#### 3.1.5. General Procedure for Target Compounds (**5a**–**5ad**)

5-(4-((5(6)-Substituted-1*H*-benzimidazol-2-yl)phenyl)-4-methyl/ethyl-4*H*-1,2,4-triazole-3-thiol (**4a**–**4f**) (0.01 mol), potassium carbonate (0.138 g, 0.01 mol) and appropriate substituted 2-bromoacetophenone derivative (0.01 mol) were dissolved in acetone. The solution was refluxed at 40 °C for 12 h. Acetone was evaporated, residue was washed with water, filtered, dried and recrystallized from ethanol.

*2-((5-(4-(1H-Benzimidazol-2-yl)phenyl)-4-methyl-4H-1,2,4-triazol-3-yl)thio)-1-phenylethan-1-one* (**5a**): FTIR (ATR, cm^−1^): 3140 (N-H), 1682 (C=O), 1193, 851, 752. ^1^H-NMR (300 MHz, DMSO-*d*_6_): δ = 3.72 (3H, s, -CH_3_), 4.94 (2H, s, -CH_2_-), 7.22–7.25 (2H, m, Benzimidazole C-H), 7.54–7.73 (4H, m, Monosubstituted Benzene C-H), 7.83 (2H, d, *J =* 8.45 Hz, Benzene C-H), 7.87 (1H, d, *J =* 8.50 Hz Benzimidazole C-H), 8.36 (2H, d, *J =* 8.45 Hz, Benzene C-H) ), 13.13 (1H, s, Benzimidazole N-H). ^13^C-NMR (75 MHz, DMSO-*d*_6_): δ = 32.50, 41.11, 112.08, 119.50, 122.81, 127.39, 128.64, 128.92, 129.32, 129.55, 131.94, 132.55, 134.25, 135.78, 150.57, 150.62, 150.80, 151.06, 154.79, 193.71. HRMS (*m*/*z*): [M + H]^+^ calcd. for C_24_H_19_N_5_OS: 426.1383; found: 426.1377.

*2-((5-(4-(1H-Benzimidazol-2-yl)phenyl)-4-methyl-4H-1,2,4-triazol-3-yl)thio)-1-(4-chlorophenyl)ethan-1-one* (**5b**): FTIR (ATR, cm^−1^): 3154 (N-H), 1672 (C=O), 1196, 812, 735. ^1^H-NMR (300 MHz, DMSO-*d*_6_): δ = 3.72 (3H, s, -CH_3_), 4.94 (2H, s, -CH_2_-), 7.19–7.28 (2H, m, Benzimidazole C-H), 7.57 (1H, d, *J =* 7.20 Hz, Benzimidazole C-H), 7.64 (2H, d, *J =* 8.60 Hz, Chlorophenyl C-H), 7.70 (1H, d, *J =* 7.20 Hz Benzimidazole C-H), 7.90 (2H, d, *J =* 8.40 Hz, Benzene C-H), 8.06 (2H, d, *J =* 8.60 Hz, Chlorophenyl C-H), 8.34 (2H, d, *J =* 8.40 Hz, Benzene C-H), 13.08 (1H, s, Benzimidazole N-H). ^13^C-NMR (75 MHz, DMSO-*d*_6_): δ = 32.50, 41.11, 111.96, 119.54, 122.38, 127.24, 128.44, 129.02, 129.24, 129.42, 130.86, 131.81, 134.46, 135.54, 139.16, 144.28, 150.81, 150.94, 155.33, 193.02. HRMS (*m*/*z*): [M + H]^+^ calcd. for C_24_H_18_N_5_ClOS: 460.0993; found: 460.0979.

*2-((5-(4-(1H-Benzimidazol-2-yl)phenyl)-4-methyl-4H-1,2,4-triazol-3-yl)thio)-1-(4-fluorophenyl)ethan-1-one* (**5c**): FTIR (ATR, cm^−1^): 3240 (N-H), 1676 (C=O), 1470, 1001, 743. ^1^H-NMR (300 MHz, DMSO-*d*_6_): δ = 3.72 (3H, s, -CH_3_), 4.58 (2H, s, -CH_2_-), 7.21–7.26 (2H, m, Benzimidazole C-H), 7.40 (2H, t, *J =* 8.80 Hz, Fluorophenyl C-H), 7.57 (1H, d, *J =* 7.05 Hz, Benzimidazole C-H), 7.70 (1H, d, *J =* 7.05 Hz, Benzimidazole C-H), 7.90 (2H, d, *J =* 8.40 Hz, Benzene C-H), 8.11–8.16 (2H, m, Fluorophenyl C-H), 8.34 (2H, d, *J =* 8.40 Hz, Benzene C-H), 13.08 (1H, s, Benzimidazole N-H). ^13^C-NMR (75 MHz, DMSO-*d*_6_): δ = 32.50, 41.14, 111.96, 116.36 (d, *J =* 22.0 Hz), 119.54, 122.38, 123.38, 127.24, 128.46, 129.23, 131.81, 132.03 (d, *J =* 9.6 Hz), 132.53 (d, *J =* 2.8 Hz), 135.55, 144.29, 150.81, 151.01, 155.32, 166.79 (d, *J =* 252.2 Hz), 192.54. HRMS (*m*/*z*): [M + H]^+^ calcd. for C_24_H_18_N_5_FOS: 444.1289; found: 444.1293.

*2-((5-(4-(1H-Benzimidazol-2-yl)phenyl)-4-methyl-4H-1,2,4-triazol-3-yl)thio)-1-(2,4-dichlorophenyl)ethan-1-one* (**5d**): FTIR (ATR, cm^−1^): 3098 (N-H), 1661 (C=O), 1472, 997, 735. ^1^H-NMR (300 MHz, DMSO-*d*_6_): δ = 3.70 (3H, s, -CH_3_), 4.81 (2H, s, -CH_2_-), 7.22–7.31 (2H, m, Benzimidazole C-H), 7.61 (1H, dd, *J =* 8.40 Hz, 2.00 Hz, Dichlorophenyl C-H), 7.64–7.70 (2H, m, Benzimidazole C-H), 7.77 (1H, d, *J =* 2.00 Hz, Dichlorophenyl C-H), 7.90 (1H, d, *J =* 8.40 Hz, Dichlorophenyl C-H), 7.92 (2H, d, *J =* 8.45 Hz, Benzene C-H), 8.36 (2H, d, *J =* 8.45 Hz, Benzene C-H). ^13^C-NMR (75 MHz, DMSO-*d*_6_): δ = 32.47, 43.20, 115.60, 123.41, 123.50, 127.50, 128.05, 128.80, 129.29, 130.59, 130.84, 131.58, 131.99, 132.10, 135.77, 137.28, 138.90, 150.48, 150.91, 155.25, 157.26, 194.96. HRMS (*m*/*z*): [M + H]^+^ calcd. for C_24_H_17_N_5_Cl_2_OS: 494.0604; found: 494.0588.

*2-((5-(4-(1H-Benzimidazol-2-yl)phenyl)-4-methyl-4H-1,2,4-triazol-3-yl)thio)-1-(2,4-difluorophenyl)ethan-1-one* (**5e**): FTIR (ATR, cm^−1^): 3121 (N-H), 1622 (C=O), 1474, 839,746. ^1^H-NMR (300 MHz, DMSO-*d*_6_): δ = 3.72 (3H, s, -CH_3_), 4.83 (2H, s, -CH_2_-), 7.22–7.31 (3H, m, Benzimidazole C-H, Difluorophenyl C-H), 7.57 (1H, td, *J =* 10.40–2.40 Hz, Difluorophenyl C-H), 7.63 (2H, br. s, Benzimidazole C-H), 7.90 (2H, d, *J =* 8.40 Hz, Benzene C-H), 8.02 (1H, td, *J =* 10.40–2.40 Hz, Difluorophenyl C-H), 8.34 (2H, d, *J =* 8.50 Hz, Benzene C-H), 13.08 (1H, s, Benzimidazole N-H). ^13^C-NMR (75 MHz, DMSO-*d*_6_): δ = 32.45, 44.34, 105.79 (t, *J =* 26.8 Hz), 111.92, 113.02 (dd, *J =* 21.4 Hz, 3.4 Hz), 119.72, 121.45, 122.97, 127.24, 129.25, 129.51, 131.81, 132.15, 133.37 (dd, *J =* 11.0 Hz, 3.0 Hz), 150.82, 150.98, 155.31, 157.37, 162.21 (dd, *J =* 255.8 Hz, 13.4 Hz), 163.91 (dd, *J =* 252.8 Hz, 12.8 Hz), 170.16, 190.37 (d, *J =* 3.9 Hz). HRMS (*m*/*z*): [M + H]^+^ calcd. for C_24_H_17_N_5_F_2_OS: 462.1195; found: 462.1178.

*2-((5-(4-(1H-Benzimidazol-2-yl)phenyl)-4-ethyl-4H-1,2,4-triazol-3-yl)thio)-1-phenylethan-1-one* (**5f**): FTIR (ATR, cm^−1^): 3127 (N-H), 1682 (C=O), 853, 746. ^1^H-NMR (300 MHz, DMSO-*d*_6_): δ = 1.29 (3H, t, *J =* 7.20 Hz, -CH_3_), 4.12 (2H, q, *J =* 7.20 Hz, -CH_2_), 5.03 (2H, s, -CH_2_-), 7.22–7.25 (2H, m, Benzimidazole C-H), 7.55–7.72 (4H, m, Monosubstituted Benzene C-H), 7.84 (2H, d, *J =* 8.45 Hz, Benzene C-H), 7.88 (1H, d, *J =* 8.50 Hz Benzimidazole C-H), 8.36 (2H, d, *J =* 8.45 Hz, Benzene C-H) ), 13.13 (1H, s, Benzimidazole N-H). ^13^C-NMR (75 MHz, DMSO-*d*_6_): δ = 13.96, 15.54, 41.24, 112.08, 119.50, 122.81, 127.39, 128.64, 128.92, 129.29, 129.55, 131.94, 132.55, 134.25, 135.78, 150.57, 150.62, 150.80, 151.06, 154.79, 193.71. HRMS (*m*/*z*): [M + H]^+^ calcd. for C_25_H_21_N_5_OS: 440.1540; found: 440.1522.

*2-((5-(4-(1H-Benzimidazol-2-yl)phenyl)-4-ethyl-4H-1,2,4-triazol-3-yl)thio)-1-(4-chlorophenyl)ethan-1-one* (**5g**): FTIR (ATR, cm^−1^): 3431 (N-H), 1680 (C=O), 814, 735. ^1^H-NMR (300 MHz, DMSO-*d*_6_): δ = 1.28 (3H, t, *J =* 7.15 Hz, -CH_3_), 4.12 (2H, q, *J =* 7.15 Hz, -CH_2_), 5.01 (2H, s, -CH_2_-), 7.25–7.29 (2H, m, Benzimidazole C-H), 7.61–7.68 (4H, m, Benzimidazole C-H, Chlorophenyl C-H), 7.86 (2H, d, *J =* 8.40 Hz, Benzene C-H), 8.07 (2H, d, *J =* 8.60 Hz, Chlorophenyl C-H), 8.36 (2H, d, *J =* 8.40 Hz, Benzene C-H), 14.03 (1H, s, Benzimidazole N-H). ^13^C-NMR (75 MHz, DMSO-*d*_6_): δ = 13.94, 15.53, 41.08, 111.96, 119.54, 123.21, 127.54, 128.88, 129.32, 129.61, 130.71, 130.85, 131.32, 132.12, 134.51, 139.16, 145.66, 150.59, 151.02, 154.75, 192.87. HRMS (*m*/*z*): [M + H]^+^ calcd. for C_25_H_20_N_5_ClOS: 474.1150; found: 474.1131.

*2-((5-(4-(1H-Benzimidazol-2-yl)phenyl)-4-ethyl-4H-1,2,4-triazol-3-yl)thio)-1-(4-fluorophenyl)ethan-1-one* (**5h**): FTIR (ATR, cm^−1^): 3191 (N-H), 1678 (C=O), 1236, 845, 741. ^1^H-NMR (300 MHz, DMSO-*d*_6_): δ = 1.29 (3H, t, *J =* 7.20 Hz, -CH_3_), 4.13 (2H, q, *J =* 7.20 Hz, -CH_2_), 5.01 (2H, s, -CH_2_-), 7.21–7.26 (2H, m, Benzimidazole C-H), 7.41 (2H, t, *J =* 8.80 Hz, Fluorobenzene C-H), 7.57 (1H, d, *J =* 7.10 Hz, Benzimidazole C-H), 7.71 (1H, d, *J =* 7.10 Hz, Benzimidazole C-H), 7.84 (2H, d, *J =* 8.45 Hz, Benzene C-H), 8.12–8.17 (2H, m, Fluorobenzene C-H), 8.34 (2H, d, *J =* 8.45 Hz, Benzene C-H) ), 13.08 (1H, s, Benzimidazole N-H). ^13^C-NMR (75 MHz, DMSO-*d*_6_): δ = 13.96, 15.54, 41.10, 111.97, 116.37 (d, *J =* 22.0 Hz), 119.56, 122.38, 123.38, 127.38, 128.63, 129.28, 131.46, 132.01 (d, *J =* 10.1 Hz), 132.57 (d, *J =* 3.1 Hz), 135.55, 144.29, 150.50, 150.79, 154.80, 165.79 (d, *J =* 252.8 Hz), 192.40. HRMS (*m*/*z*): [M + H]^+^ calcd. for C_25_H_20_N_5_FOS: 458.1445; found: 458.1428.

*2-((5-(4-(1H-Benzimidazol-2-yl)phenyl)-4-ethyl-4H-1,2,4-triazol-3-yl)thio)-1-(2,4-dichlorophenyl)ethan-1-one* (**5i**): FTIR (ATR, cm^−1^): 3146 (N-H), 1686 (C=O), 1141, 853, 743. ^1^H-NMR (300 MHz, DMSO-*d*_6_): δ = 1.25 (3H, t, *J =* 7.15 Hz, -CH_3_), 4.07 (2H, q, *J =* 7.15 Hz, -CH_2_), 4.86 (2H, s, -CH_2_-), 7.22–7.26 (2H, m, Benzimidazole C-H), 7.61 (1H, dd, *J =* 8.40 Hz, 1.90 Hz, Dichlorophenyl C-H), 7.64 (2H, br. s, Benzimidazole C-H), 7.77 (1H, d, *J =* 1.90 Hz, Dichlorophenyl C-H), 7.84 (2H, d, *J =* 8.40 Hz, Benzene C-H), 7.90 (1H, d, *J =* 8.40 Hz, Dichlorophenyl C-H), 8.35 (2H, d, *J =* 8.40 Hz, Benzene C-H), 13.08 (1H, s, Benzimidazole N-H). ^13^C-NMR (75 MHz, DMSO-*d*_6_): δ = 13.96, 15.47, 43.06, 119.22, 122.89, 123.11, 127.39, 128.05, 128.53, 129.29, 130.58, 130.83, 131.52, 131.98, 132.08, 135.83, 137.27, 138.86, 150.25, 150.78, 154.87, 157.26, 194.95. HRMS (*m*/*z*): [M + H]^+^ calcd. for C_25_H_19_N_5_Cl_2_OS: 508.0760; found: 508.0755.

*2-((5-(4-(1H-Benzimidazol-2-yl)phenyl)-4-ethyl-4H-1,2,4-triazol-3-yl)thio)-1-(2,4-difluorophenyl)ethan-1-one* (**5j**): FTIR (ATR, cm^−1^): 3422 (N-H), 1672 (C=O), 858, 746. ^1^H-NMR (300 MHz, DMSO-*d*_6_): δ = 1.28 (3H, t, *J =* 7.20 Hz, -CH_3_), 4.11 (2H, q, *J =* 7.20 Hz, -CH_2_), 4.80 (2H, s, -CH_2_-), 7.22–7.32 (3H, m, Benzimidazole C-H, Difluorobenzene C-H), 7.46–7.54 (1H, m, Difluorobenzene C-H), 7.63 (2H, br. s, Benzimidazole C-H), 7.84 (2H, d, *J =* 8.40 Hz, Benzene C-H), 8.00–8.07 (1H, m, Difluorobenzene C-H), 8.34 (2H, d, *J =* 8.40 Hz, Benzene C-H), 13.08 (1H, s, Benzimidazole N-H). ^13^C-NMR (75 MHz, DMSO-*d*_6_): δ = 13.96, 15.50, 44.30, 105.64 (t, *J =* 26.5 Hz), 111.92, 113.00 (dd, *J =* 21.5 Hz, 3.3 Hz), 119.69, 121.45, 122.99, 127.20, 129.27, 129.62, 131.80, 132.17, 133.39 (dd, *J =* 11.2 Hz, 2.8 Hz), 150.82, 151.08, 155.38, 157.32, 162.23 (dd, *J =* 253.4 Hz, 13.9 Hz), 163.88 (dd, *J =* 251.2 Hz, 12.6 Hz), 170.12, 190.32 (d, *J =* 3.7 Hz). HRMS (*m*/*z*): [M + H]^+^ calcd. for C_25_H_19_N_5_F_2_OS: 476.1351; found: 476.1343.

*2-((5-(4-(6-Chloro-1H-benzimidazol-2-yl)phenyl)-4-methyl-4H-1,2,4-triazol-3-yl)thio)-1-phenylethan-1-one* (**5k**): FTIR (ATR, cm^−1^): 3113 (N-H), 1678 (C=O), 1558, 849, 748. ^1^H-NMR (300 MHz, DMSO-*d*_6_): δ = 3.72 (3H, s, -CH_3_), 4.96 (2H, s, -CH_2_-), 7.26 (1H, dd, *J =* 8.55 Hz, 1.65 Hz, Benzimidazole C-H), 7.54–7.72 (4H, m, Monosubstituted Benzene C-H, Benzimidazole C-H), 7.91 (2H, d, *J =* 8.30 Hz, Benzene C-H), 8.05 (2H, d, *J =* 7.35 Hz Monosubstituted Benzene C-H), 8.33 (2H, d, *J =* 8.30 Hz, Benzene C-H). ^13^C-NMR (75 MHz, DMSO-*d*_6_): δ = 32.50, 41.28, 115.17, 116.06, 123.19, 127.21, 127.43, 128.47, 128.84, 128.92, 129.30, 129.46, 130.82, 131.25, 134.25, 135.73, 151.15, 152.22, 155.22, 193.83. HRMS (*m*/*z*): [M + H]^+^ calcd. for C_24_H_18_N_5_ClOS: 460.0993; found: 460.0979.

*2-((5-(4-(6-Chloro-1H-benzimidazol-2-yl)phenyl)-4-methyl-4H-1,2,4-triazol-3-yl)thio)-1-(4-chlorophenyl)ethan-1-one* (**5l**): FTIR (ATR, cm^−1^): 3136 (N-H), 1674 (C=O), 1092, 851, 710. ^1^H-NMR (300 MHz, DMSO-*d*_6_): δ = 3.71 (3H, s, -CH_3_), 4.94 (2H, s, -CH_2_-), 7.25 (2H, dd, *J =* 8.55 Hz, 1.75 Hz, Benzimidazole C-H), 7.62–7.68 (3H, m, Benzimidazole C-H, Chlorophenyl C-H), 7.68 (1H, d, *J =* 1.75 Hz Benzimidazole C-H), 7.91 (2H, d, *J =* 8.30 Hz, Benzene C-H), 8.05 (2H, d, *J =* 8.50 Hz, Chlorophenyl C-H), 8.33 (2H, d, *J =* 8.30 Hz, Benzene C-H). ^13^C-NMR (75 MHz, DMSO-*d*_6_): δ = 32.51, 41.12, 115.51, 116.55, 123.10, 127.12, 127.41, 128.36, 128.76, 128.95, 129.26, 129.41, 130.85, 131.42, 134.46, 139.15, 150.99, 152.33, 155.26, 193.00. HRMS (*m*/*z*): [M + H]^+^ calcd. for C_24_H_17_N_5_Cl_2_OS: 494.0604; found: 494.0594.

*2-((5-(4-(6-Chloro-1H-benzimidazol-2-yl)phenyl)-4-methyl-4H-1,2,4-triazol-3-yl)thio)-1-(4-fluorophenyl)ethan-1-one* (**5m**): FTIR (ATR, cm^−1^): 3069 (N-H), 1672 (C=O), 1472, 1194, 839, 735. ^1^H-NMR (300 MHz, DMSO-*d*_6_): δ = 3.72 (3H, s, -CH_3_), 4.95 (2H, s, -CH_2_-), 7.27 (1H, d, *J =* 8.70 Hz, Benzimidazole C-H), 7.40 (2H, t, *J =* 8.80 Hz, Fluorophenyl C-H), 7.59 (1H, s, Benzimidazole C-H), 7.75 (1H, d, *J =* 8.70 Hz, Benzimidazole C-H), 7.90 (2H, d, *J =* 8.40 Hz, Benzene C-H), 8.11–8.16 (2H, m, Fluorophenyl C-H), 8.34 (2H, d, *J =* 8.40 Hz, Benzene C-H), 13.32 (1H, s, Benzimidazole N-H). ^13^C-NMR (75 MHz, DMSO-*d*_6_): δ = 32.51, 41.14, 113.31, 116.36 (d, *J =* 22.0 Hz), 118.90, 122.78, 123.47, 127.42, 128.83, 129.26, 131.32, 132.02 (d, *J =* 9.6 Hz), 132.53 (d, *J =* 3.1 Hz), 134.38, 136.29, 145.19, 151.07, 155.25, 165.78 (d, *J =* 252.6 Hz), 192.54. HRMS (*m*/*z*): [M + H]^+^ calcd. for C_24_H_17_N_5_ClFOS: 478.0899; found: 478.0898.

*2-((5-(4-(6-Chloro-1H-Benzimidazol-2-yl)phenyl)-4-methyl-4H-1,2,4-triazol-3-yl)thio)-1-(2,4-dichlorophenyl)ethan-1-one* (**5n**): FTIR (ATR, cm^−1^): 3086 (N-H), 1663 (C=O), 1472, 1063, 839, 708. ^1^H-NMR (300 MHz, DMSO-*d*_6_): δ = 3.68 (3H, s, -CH_3_), 4.81 (2H, s, -CH_2_-), 7.25 (1H, dd, *J =* 8.50 Hz, 1.35 Hz Benzimidazole C-H), 7.59 (1H, dd, *J =* 8.40 Hz, 1.90 Hz, Dichlorophenyl C-H), 7.64–7.73 (2H, m, Benzimidazole C-H), 7.75 (1H, d, *J =* 1.90 Hz, Dichlorophenyl C-H), 7.88–7.92 (3H, m, Dichlorophenyl C-H, Benzene C-H), 8.33 (2H, d, *J =* 8.35 Hz, Benzene C-H), 13.32 (1H, s, Benzimidazole N-H). ^13^C-NMR (75 MHz, DMSO-*d*_6_): δ = 32.46, 43.22, 112.09, 113.24, 118.88, 120.90, 123.25, 127.41, 128.03, 128.70, 129.25, 130.58, 131.36, 131.99, 132.08, 135.75, 137.28, 144.58, 150.87, 152.24, 155.27, 194.93. HRMS (*m*/*z*): [M + H]^+^ calcd. for C_24_H_16_N_5_Cl_3_OS: 528.0214; found: 528.0221.

*2-((5-(4-(6-Chloro-1H-benzimidazol-2-yl)phenyl)-4-methyl-4H-1,2,4-triazol-3-yl)thio)-1-(2,4-difluorophenyl)ethan-1-one* (**5o**): FTIR (ATR, cm^−1^): 3119 (N-H), 1672 (C=O), 1472, 1229, 849, 708. ^1^H-NMR (300 MHz, DMSO-*d*_6_): δ = 3.72 (3H, s, -CH_3_), 4.84 (2H, s, -CH_2_-), 7.22–7.31 (2H, m, Benzimidazole C-H, Difluorophenyl C-H), 7.48 (1H, td, *J =* 10.40–2.20 Hz, Difluorophenyl C-H), 7.59 (1H, s, Benzimidazole C-H), 7.70 (1H, d, *J =* 8.40 Hz, Benzimidazole C-H), 7.91 (2H, d, *J =* 8.35 Hz, Benzene C-H), 7.98–8.06 (1H, m, Difluorophenyl C-H), 8.33 (2H, d, *J =* 8.35 Hz, Benzene C-H), 13.31 (1H, s, Benzimidazole N-H). ^13^C-NMR (75 MHz, DMSO-*d*_6_): δ = 32.44, 43.86, 105.65 (t, *J =* 26.6 Hz), 111.92, 113.02 (dd, *J =* 21.8 Hz, 3.2 Hz), 119.72, 121.45, 122.97, 127.24, 129.38, 130.68, 131.93, 133.29 (dd, *J =* 11.2 Hz, 2.8 Hz), 150.94, 152.59, 154.24, 160.08 (dd, *J =* 255.2 Hz, 13.0 Hz), 164.16 (dd, *J =* 252.6 Hz, 12.4 Hz), 168.12, 192.23 (d, *J =* 3.6 Hz). HRMS (*m*/*z*): [M + H]^+^ calcd. for C_24_H_16_N_5_ClF_2_OS: 496.0805; found: 496.0814.

*2-((5-(4-(6-Chloro-1H-benzimidazol-2-yl)phenyl)-4-ethyl-4H-1,2,4-triazol-3-yl)thio)-1-phenylethan-1-one* (**5p**): FTIR (ATR, cm^−1^): 3252 (N-H), 1666 (C=O), 853, 754. ^1^H-NMR (300 MHz, DMSO-*d*_6_): δ = 1.28 (3H, t, *J =* 7.20 Hz, -CH_3_), 4.13 (2H, q, *J =* 7.20 Hz, -CH_2_), 5.03 (2H, s, -CH_2_-), 7.27 (1H, dd, *J =* 8.60 Hz, 2.00 Hz, Benzimidazole C-H), 7.55–7.72 (4H, m, Monosubstituted benzene C-H, Benzimidazole C-H), 7.86 (2H, d, *J =* 8.50 Hz, Benzene C-H), 7.88 (1H, d, *J =* 8.50 Hz Benzimidazole C-H), 8.04–8.08 (2H, m, Monosubstituted benzene C-H), 8.34 (2H, d, *J =* 8.50 Hz, Benzene C-H). ^13^C-NMR (75 MHz, DMSO-*d*_6_): δ = 13.96, 15.52, 41.23, 115.53, 115.91, 123.31, 127.33, 127.61, 128.53, 128.78, 128.91, 129.08, 129.32, 130.66, 131.19, 134.26, 135.77, 150.68, 152.12, 154.68, 193.69. HRMS (*m*/*z*): [M + H]^+^ calcd. for C_25_H_20_N_5_ClOS: 474.1150; found: 474.1142.

*2-((5-(4-(6-Chloro-1H-benzimidazol-2-yl)phenyl)-4-ethyl-4H-1,2,4-triazol-3-yl)thio)-1-(4-chlorophenyl)ethan-1-one* (**5q**): FTIR (ATR, cm^−1^): 3202 (N-H), 1674 (C=O), 1589, 856, 773. ^1^H-NMR (300 MHz, DMSO-*d*_6_): δ = 1.28 (3H, t, *J =* 7.15 Hz, -CH_3_), 4.12 (2H, q, *J =* 7.15 Hz, -CH_2_), 5.00 (2H, s, -CH_2_-), 7.25 (1H, dd, *J =* 8.50 Hz, 1.40 Hz, Benzimidazole C-H), 7.63 (2H, d, *J =* 8.60 Hz, Chlorophenyl C-H), 7.57 (1H, d, *J =* 7.20 Hz, Benzimidazole C-H), 7.68 (1H, br. s, Benzimidazole C-H), 7.85 (2H, d, *J =* 8.40 Hz, Benzene C-H), 8.06 (2H, d, *J =* 8.60 Hz, Chlorophenyl C-H), 8.34 (2H, d, *J =* 8.40 Hz, Benzene C-H), 13.28 (1H, s, Benzimidazole N-H). ^13^C-NMR (75 MHz, DMSO-*d*_6_): δ = 13.96, 15.52, 41.09, 113.26, 118.89, 123.23, 127.05, 127.54, 128.26, 128.69, 128.95, 129.30, 129.41, 130.83, 131.45, 134.49, 139.16, 150.51, 152.18, 154.74, 193.84. HRMS (*m*/*z*): [M + H]^+^ calcd. for C_25_H_19_N_5_Cl_2_OS: 508.0760; found: 508.0781.

*2-((5-(4-(6-Chloro-1H-benzimidazol-2-yl)phenyl)-4-ethyl-4H-1,2,4-triazol-3-yl)thio)-1-(4-fluorophenyl)ethan-1-one* (**5r**): FTIR (ATR, cm^−1^): 3111 (N-H), 1678 (C=O), 1196, 847, 795. ^1^H-NMR (300 MHz, DMSO-*d*_6_): δ = 1.28 (3H, t, *J =* 7.20 Hz, -CH_3_), 4.12 (2H, q, *J =* 7.20 Hz, -CH_2_), 5.01 (2H, s, -CH_2_-), 7.27 (1H, d, *J =* 8.60 Hz, Benzimidazole C-H), 7.41 (2H, t, *J =* 8.80 Hz, Fluorobenzene C-H), 7.59 (1H, s, Benzimidazole C-H), 7.71 (1H, d, *J =* 8.60 Hz, Benzimidazole C-H), 7.85 (2H, d, *J =* 8.35 Hz, Benzene C-H), 8.12–8.19 (2H, m, Fluorobenzene C-H), 8.33 (2H, d, *J =* 8.35 Hz, Benzene C-H), 13.29 (1H, s, Benzimidazole N-H). ^13^C-NMR (75 MHz, DMSO-*d*_6_): δ = 13.96, 15.53, 41.10, 111.68, 116.37 (d, *J =* 22.1 Hz), 118.93, 122.80, 123.47, 127.56, 129.00, 129.31, 131.44, 132.01 (d, *J =* 9.6 Hz), 132.56 (d, *J =* 2.8 Hz), 134.37, 136.29, 145.19, 150.57, 154.72, 165.79 (d, *J =* 252.6 Hz), 192.38. HRMS (*m*/*z*): [M + H]^+^ calcd. for C_25_H_19_N_5_ClFOS: 492.1056; found: 492.1036.

*2-((5-(4-(6-Chloro-1H-benzimidazol-2-yl)phenyl)-4-ethyl-4H-1,2,4-triazol-3-yl)thio)-1-(2,4-dichlorophenyl)ethan-1-one* (**5s**): FTIR (ATR, cm^−1^): 3157 (N-H), 1709 (C=O), 1580, 856, 746. ^1^H-NMR (300 MHz, DMSO-*d*_6_): δ = 1.27 (3H, t, *J =* 7.15 Hz, -CH_3_), 4.11 (2H, q, *J =* 7.15 Hz, -CH_2_), 4.89 (2H, s, -CH_2_-), 6.86 (1H, d, *J =* 8.25 Hz, Dichlorophenyl C-H), 7.25 (1H, dd, *J =* 8.55 Hz, 1.85 Hz, Benzimidazole C-H), 7.41 (1H, d, *J =* 2.10 Hz, Dichlorophenyl C-H), 7.46 (1H, dd, *J =* 8.25 Hz, 2.10 Hz, Dichlorophenyl C-H), 7.65 (2H, br. s, Benzimidazole C-H), 7.85 (2H, d, *J =* 8.45 Hz, Benzene C-H), 8.33 (2H, d, *J =* 8.45 Hz, Benzene C-H). ^13^C-NMR (75 MHz, DMSO-*d*_6_): δ = 13.96, 15.53, 41.08, 111.89, 115.67, 118.88, 122.49, 123.19, 127.54, 128.05, 129.02, 129.32, 130.59, 131.42, 131.90, 132.10, 135.77, 137.28, 145.84, 150.86, 152.22, 154.66, 191.72. HRMS (*m*/*z*): [M + H]^+^ calcd. for C_25_H_18_N_5_Cl_3_OS: 542.0370; found: 542.0369.

*2-((5-(4-(6-Chloro-1H-benzimidazol-2-yl)phenyl)-4-ethyl-4H-1,2,4-triazol-3-yl)thio)-1-(2,4-difluorophenyl)ethan-1-one* (**5t**): FTIR (ATR, cm^−1^): 3117 (N-H), 1678 (C=O), 1186, 853, 716. ^1^H-NMR (300 MHz, DMSO-*d*_6_): δ = 1.28 (3H, t, *J =* 7.20 Hz, -CH_3_), 4.11 (2H, q, *J =* 7.20 Hz, -CH_2_), 4.90 (2H, s, -CH_2_-), 7.24–7.32 (2H, m, Benzimidazole C-H, Difluorophenyl C-H), 7.49 (1H, td, *J =* 10.45 Hz, 2.45 Hz, Difluorophenyl C-H), 7.64 (2H, br. s, Benzimidazole C-H), 7.85 (2H, d, *J =* 8.50 Hz, Benzene C-H), 7.98–8.06 (1H, m, Difluorophenyl C-H), 8.33 (2H, d, *J =* 8.50 Hz, Benzene C-H), 13.28 (1H, s, Benzimidazole N-H). ^13^C-NMR (75 MHz, DMSO-*d*_6_): δ = 13.96, 15.49, 44.30, 105.80 (t, *J =* 26.7 Hz), 111.86, 113.05 (dd, *J =* 21.6 Hz, 3.4 Hz), 119.67, 121.31, 123.06, 127.54, 129.33, 130.62, 131.45, 133.38 (dd, *J =* 10.6–3.0 Hz), 150.49, 152.86, 154.74, 162.21 (dd, *J =* 255.8 Hz, 13.4 Hz), 163.91 (dd, *J =* 252.8 Hz, 12.8 Hz), 170.16, 190.37 (d, *J =* 4.3 Hz). HRMS (*m*/*z*): [M + H]^+^ calcd. for C_25_H_18_N_5_ClF_2_OS: 510.0961; found: 510.0948.

*2-((5-(4-(6-Fluoro-1H-benzimidazol-2-yl)phenyl)-4-methyl-4H-1,2,4-triazol-3-yl)thio)-1-phenylethan-1-one* (**5u**): FTIR (ATR, cm^−1^): 3171 (N-H), 1678 (C=O), 1472, 1136, 849, 752. ^1^H-NMR (300 MHz, DMSO-*d*_6_): δ = 3.72 (3H, s, -CH_3_), 4.96 (2H, s, -CH_2_-), 7.06–7.12 (1H, m, Benzimidazole C-H), 7.44 (1H, d, *J =* 7.30 Hz, Benzimidazole C-H), 7.53–7.72 (4H, m, Monosubstituted Benzene C-H, Benzimidazole C-H), 7.92 (2H, d, *J =* 8.30 Hz, Benzene C-H), 8.04 (2H, d, *J =* 8.25 Hz, Monosubstituted Benzene C-H), 8.34 (2H, d, *J =* 8.30 Hz, Benzene C-H), 13.23 (1H, s, Benzimidazole N-H). ^13^C-NMR (75 MHz, DMSO-*d*_6_): δ = 32.45, 41.28, 99.39 (d, *J =* 26.6 Hz), 105.45 (d, *J =* 23.3 Hz), 112.68 (d, *J =* 10.3 Hz), 120.44, 127.39 (d, *J =* 9.1 Hz), 128.91, 129.31, 131.67, 132.25, 134.26, 135.77, 140.97, 150.61, 151.80 (d, *J =* 2.8 Hz), 152.54, 154.74, 159.03 (d, *J =* 232.6 Hz), 193.84. HRMS (*m*/*z*): [M + H]^+^ calcd. for C_24_H_18_N_5_FOS: 444.1289; found: 444.1291.

*1-(4-Chlorophenyl)-2-((5-(4-(6-fluoro-1H-benzimidazol-2-yl)phenyl)-4-methyl-4H-1,2,4-triazol-3-yl)thio)ethan-1-one* (**5v**): FTIR (ATR, cm^−1^): 3100 (N-H), 1666 (C=O), 1445, 1138, 843, 708. ^1^H-NMR (300 MHz, DMSO-*d*_6_): δ = 3.72 (3H, s, -CH_3_), 5.01 (2H, s, -CH_2_-), 7.08–7.15 (1H, m, Benzimidazole C-H), 7.44 (1H, br. s, Benzimidazole C-H), 7.50–7.59 (1H, m, Benzimidazole C-H), 7.63 (2H, d, *J =* 8.55 Hz, Chlorophenyl C-H), 7.84 (2H, d, *J =* 8.40 Hz, Benzene C-H), 8.06 (2H, d, *J =* 8.60 Hz, Chlorophenyl C-H), 8.35 (2H, d, *J =* 8.40 Hz, Benzene C-H), 13.23 (1H, s, Benzimidazole N-H). ^13^C-NMR (75 MHz, DMSO-*d*_6_): δ = 32.43, 41.10, 99.39 (d, *J =* 26.6 Hz), 105.45 (d, *J =* 23.3 Hz), 112.68 (d, *J =* 10.3 Hz), 123.23, 127.54 (d, *J =* 9.1 Hz), 128.95, 129.30, 129.41, 130.83, 131.45, 134.49, 139.16, 150.51, 151.80 (d, *J =* 2.8 Hz), 152.18, 154.74, 159.03 (d, *J =* 232.6 Hz), 193.84. HRMS (*m*/*z*): [M + H]^+^ calcd. for C_24_H_17_N_5_ClFOS: 478.0899; found: 478.0906.

*2-((5-(4-(6-Fluoro-1H-benzimidazol-2-yl)phenyl)-4-methyl-4H-1,2,4-triazol-3-yl)thio)-1-(4-fluorophenyl)ethan-1-one* (**5w**): FTIR (ATR, cm^−1^): 3176 (N-H), 1677 (C=O), 1192, 847, 753. ^1^H-NMR (300 MHz, DMSO-*d*_6_): δ = 3.72 (3H, s, -CH_3_), 4.94 (2H, s, -CH_2_-), 7.09 (1H, d, *J =* 8.70 Hz, Benzimidazole C-H), 7.40 (2H, t, *J =* 8.40 Hz, Fluorophenyl C-H), 7.48–7.55 (1H, m, Benzimidazole C-H), 7.70 (1H, br. s, Benzimidazole C-H), 7.90 (2H, d, *J =* 7.60 Hz, Benzene C-H), 8.11–8.11 (2H, m, Fluorophenyl C-H), 8.32 (2H, d, *J =* 7.60 Hz, Benzene C-H), 13.22 (1H, s, Benzimidazole N-H). ^13^C-NMR (75 MHz, DMSO-*d*_6_): δ = 32.49, 41.14, 98.32 (d, *J =* 25.5 Hz), 104.95 (d, *J =* 23.8 Hz), 113.28 (d, *J =* 10.5 Hz), 116.36 (d, *J =* 22.0 Hz), 127.26 (d, *J =* 8.5 Hz), 128.89, 129.68, 131.32, 132.02 (d, *J =* 9.4 Hz), 132.53 (d, *J =* 3.0 Hz), 134.38, 136.29, 151.04, 151.99 (d, *J =* 2.8 Hz), 155.28, 159.03 (d, *J =* 236.8 Hz), 165.78 (d, *J =* 251.2 Hz), 192.53. HRMS (*m*/*z*): [M + H]^+^ calcd. for C_24_H_17_N_5_F_2_OS: 462.1195; found: 462.1172.

*1-(2,4-Dichlorophenyl)-2-((5-(4-(6-fluoro-1H-benzimidazol-2-yl)phenyl)-4-methyl-4H-1,2,4-triazol-3-yl)thio)ethan-1-one* (**5x**): FTIR (ATR, cm^−1^): 3161 (N-H), 1665 (C=O), 1136, 835, 708. ^1^H-NMR (300 MHz, DMSO-*d*_6_): δ = 3.68 (3H, s, -CH_3_), 4.80 (2H, s, -CH_2_-), 7.07–7.13 (1H, m, Dichlorophenyl C-H), 7.44 (1H, d, *J =* 7.70 Hz, Benzimidazole C-H), 7.59–7.66 (2H, m, Dichlorophenyl C-H, Benzimidazole C-H), 7.76 (1H, s, Benzimidazole C-H), 7.88–7.91 (3H, m, Benzene C-H, Dichlorophenyl C-H), 8.33 (2H, d, *J =* 8.20 Hz, Benzene C-H). ^13^C-NMR (75 MHz, DMSO-*d*_6_): δ = 32.46, 43.21, 99.39 (d, *J =* 26.6 Hz), 105.45 (d, *J =* 23.3 Hz), 110.28, 111.42, 112.68 (d, *J =* 10.3 Hz), 122.49, 127.54 (d, *J =* 9.1 Hz), 128.05, 129.39, 130.59, 135.77, 137.28, 138.90, 145.84, 150.86, 151.80 (d, *J =* 2.8 Hz), 152.22, 154.66, 159.03 (d, *J =* 232.6 Hz), 194.95. HRMS (*m*/*z*): [M + H]^+^ calcd. for C_24_H_16_N_5_Cl_2_FOS: 512.0509; found: 512.0510.

*1-(2,4-Difluorophenyl)-2-((5-(4-(6-fluoro-1H-benzimidazol-2-yl)phenyl)-4-methyl-4H-1,2,4-triazol-3-yl)thio)ethan-1-one* (**5y**): FTIR (ATR, cm^−1^): 3190 (N-H), 1682 (C=O), 1186, 847, 710. ^1^H-NMR (300 MHz, DMSO-*d*_6_): δ = 3.71 (3H, s, -CH_3_), 4.84 (2H, s, -CH_2_-), 7.09 (1H, br. s, Benzimidazole C-H), 7.25–7.31 (1H, m, Difluorophenyl C-H), 7.44–7.48 (1H, m, Difluorophenyl C-H), 7.51 (1H, br. s, Benzimidazole C-H), 7.69 (1H, br. s, Benzimidazole -C-H), 7.90 (2H, d, *J =* 8.20 Hz, Benzene C-H), 7.98–8.06 (1H, m, Difluorophenyl C-H), 8.32 (2H, d, *J =* 8.20 Hz, Benzene C-H), 13.20 (1H, s, Benzimidazole N-H). ^13^C-NMR (75 MHz, DMSO-*d*_6_): δ = 32.48, 46.21, 99.39 (d, *J =* 26.6 Hz), 105.10 (d, *J =* 23.3 Hz), 105.92 (t, *J =* 26.8 Hz), 112.68 (d, *J =* 10.3 Hz), 113.02 (dd, *J =* 21.4 Hz, 3.4 Hz), 127.24, 127.54 (d, *J =* 9.1 Hz), 129.25, 131.81, 133.37 (dd, *J =* 11.0 Hz, 3.0 Hz), 134.42, 137.16, 150.90 (d, *J =* 12.2 Hz), 151.06, 151.80 (d, *J =* 2.8 Hz), 155.31, 159.03 (d, *J =* 232.6 Hz), 162.21 (dd, *J =* 255.8 Hz, 13.4 Hz), 163.91 (dd, *J =* 252.8 Hz, 12.8 Hz), 190.37 (d, *J =* 3.9 Hz). HRMS (*m*/*z*): [M + H]^+^ calcd. for C_24_H_16_N_5_F_3_OS: 480.1100; found: 480.1100.

*2-((4-Ethyl-5-(4-(6-fluoro-1H-benzimidazol-2-yl)phenyl)-4H-1,2,4-triazol-3-yl)thio)-1-phenylethan-1-one* (**5z**): FTIR (ATR, cm^−1^): 3184 (N-H), 1678 (C=O), 849, 756. ^1^H-NMR (300 MHz, DMSO-*d*_6_): δ = 1.28 (3H, t, *J =* 7.10 Hz, -CH_3_), 4.12 (2H, q, *J =* 7.10 Hz, -CH_2_), 5.03 (2H, s, -CH_2_-), 7.05–7.15 (1H, m, Benzimidazole C-H), 7.36 (1H, m, Benzimidazole C-H), 7.50 (1H, m, Benzimidazole C-H), 7.57 (2H, t, *J =* 7.60 Hz, Monosubstituted Benzene C-H), 7.70 (1H, t, *J =* 7.60 Hz, Monosubstituted Benzene C-H), 7.84 (2H, d, *J =* 7.90 Hz, Benzene C-H), 8.05 (2H, d, *J =* 7.60 Hz, Monosubstituted Benzene C-H), 8.33 (2H, d, *J =* 7.90 Hz, Benzene C-H), 13.21 (1H, s, Benzimidazole N-H). ^13^C-NMR (75 MHz, DMSO-*d*_6_): δ = 13.96, 15.52, 41.23, 98.32 (d, *J =* 26.9 Hz), 104.96 (d, *J =* 23.3 Hz), 112.68 (d, *J =* 10.3 Hz), 120.44, 127.39 (d, *J =* 9.1 Hz), 128.91, 129.31, 131.67, 132.25, 134.26, 135.77, 140.97, 150.61, 151.80 (d, *J =* 2.8 Hz), 152.54, 154.74, 159.03 (d, *J =* 232.6 Hz), 193.71. HRMS (*m*/*z*): [M + H]^+^ calcd. for C_25_H_20_N_5_FOS: 458.1445; found: 458.1442.

*1-(4-Chlorophenyl)-2-((4-ethyl-5-(4-(6-fluoro-1H-benzimidazol-2-yl)phenyl)-4H-1,2,4-triazol-3-yl)thio)ethan-1-one* (**5aa**): FTIR (ATR, cm^−1^): 3100 (N-H), 1666 (C=O), 1445, 1138, 843, 708. ^1^H-NMR (300 MHz, DMSO-*d*_6_): δ = 1.28 (3H, t, *J =* 7.05 Hz, -CH_3_), 4.12 (2H, q, *J =* 7.05 Hz, -CH_2_), 5.00 (2H, s, -CH_2_-), 7.06–7.13 (1H, m, Benzimidazole C-H), 7.41 (1H, br. s, Benzimidazole C-H), 7.50–7.59 (1H, m, Benzimidazole C-H), 7.63 (2H, d, *J =* 8.60 Hz, Chlorophenyl C-H), 7.84 (2H, d, *J =* 8.35 Hz, Benzene C-H), 8.06 (2H, d, *J =* 8.60 Hz, Chlorophenyl C-H), 8.33 (2H, d, *J =* 8.35 Hz, Benzene C-H), 13.23 (1H, s, Benzimidazole N-H). ^13^C-NMR (75 MHz, DMSO-*d*_6_): δ = 13.96, 15.52, 41.10, 99.23 (d, *J =* 26.6 Hz), 105.58 (d, *J =* 23.3 Hz), 113.26 (d, *J =* 10.3 Hz), 123.23, 127.54 (d, *J =* 9.0 Hz), 128.51, 129.30, 129.41, 130.61, 131.33, 134.81, 139.16, 150.51, 151.80 (d, *J =* 2.6 Hz), 152.18, 154.74, 162.75 (d, *J =* 232.8 Hz), 193.84. HRMS (*m*/*z*): [M + H]^+^ calcd. for C_25_H_19_N_5_ClFOS: 492.1056; found: 492.1061.

*2-((4-Ethyl-5-(4-(6-fluoro-1H-benzimidazol-2-yl)phenyl)-4H-1,2,4-triazol-3-yl)thio)-1-(4-fluorophenyl)ethan-1-one* (**5ab**): FTIR (ATR, cm^−1^): 3174 (N-H), 1678 (C=O), 1443, 1192, 837, 716. ^1^H-NMR (300 MHz, DMSO-*d*_6_): δ = 1.28 (3H, t, *J =* 7.20 Hz, -CH_3_), 4.12 (2H, q, *J =* 7.20 Hz, -CH_2_), 5.01 (2H, s, -CH_2_-), 7.09 (1H, td, *J =* 9.45 Hz, Benzimidazole C-H), 7.37–7.47 (3H, m, Fluorophenyl C-H, Benzimidazole C-H), 7.63 (1H, br. s, Benzimidazole C-H), 7.84 (2H, d, *J =* 8.45 Hz, Benzene C-H), 8.11–8.18 (2H, m, Fluorophenyl C-H), 8.32 (2H, d, *J =* 8.45 Hz, Benzene C-H), 13.21 (1H, s, Benzimidazole N-H). ^13^C-NMR (75 MHz, DMSO-*d*_6_): δ = 13.96, 15.53, 41.09, 100.02 (d, *J =* 25.4 Hz), 106.13 (d, *J =* 23.0 Hz), 113.31 (d, *J =* 10.3 Hz), 117.18 (d, *J =* 21.6 Hz), 127.36 (d, *J =* 9.0 Hz), 128.83, 129.28, 131.28, 131.97 (d, *J =* 9.2 Hz), 132.79 (d, *J =* 2.9 Hz), 134.16, 137.03, 150.84, 151.80 (d, *J =* 2.6 Hz), 154.64, 160.12 (d, *J =* 232.8 Hz), 166.24 (d, *J =* 252.2 Hz), 192.53. HRMS (*m*/*z*): [M + H]^+^ calcd. for C_25_H_19_N_5_F_2_OS: 476.1351; found: 476.1347.

*1-(2,4-Dichlorophenyl)-2-((4-ethyl-5-(4-(6-fluoro-1H-benzimidazol-2-yl)phenyl)-4H-1,2,4-triazol-3-yl)thio)ethan-1-one* (**5ac**): FTIR (ATR, cm^−1^): 3161 (N-H), 1688 (C=O), 1443, 1138, 851, 714. ^1^H-NMR (300 MHz, DMSO-*d*_6_): δ = 1.25 (3H, t, *J =* 7.15 Hz, -CH_3_), 4.08 (2H, q, *J =* 7.15 Hz, -CH_2_), 4.86 (2H, s, -CH_2_-), 7.06–7.13 (1H, m, Benzimidazole C-H), 7.49–7.59 (1H, m, Benzimidazole C-H), 7.61 (1H, dd, *J =* 8.40 Hz, 1.90 Hz, Dichlorophenyl C-H), 7.69–7.73 (1H, m, Benzimidazole C-H), 7.77 (1H, d, *J =* 1.90 Hz, Dichlorophenyl C-H), 7.84 (2H, d, *J =* 7.90 Hz, Benzene C-H), 7.90 (1H, d, *J =* 8.40 Hz, Dichlorophenyl C-H), 8.32 (2H, d, *J =* 7.90 Hz, Benzene C-H), 13.21 (1H, s, Benzimidazole N-H). ^13^C-NMR (75 MHz, DMSO-*d*_6_): δ = 13.96, 15.46, 43.05, 98.34 (d, *J =* 27.4 Hz), 104.95 (d, *J =* 22.8 Hz), 110.45, 111.32, 112.68 (d, *J =* 10.3 Hz), 120.44, 127.39 (d, *J =* 8.7 Hz), 128.05, 129.32, 130.57, 135.83, 137.27, 140.97, 144.74, 150.28, 151.77 (d, *J =* 3.0 Hz), 152.52, 154.82, 159.28 (d, *J =* 235.8 Hz), 194.94. HRMS (*m*/*z*): [M + H]^+^ calcd. for C_25_H_18_N_5_Cl_2_FOS: 526.0666; found: 526.0658.

*1-(2,4-Difluorophenyl)-2-((4-ethyl-5-(4-(6-fluoro-1H-benzimidazol-2-yl)phenyl)-4H-1,2,4-triazol-3-yl)thio)ethan-1-one* (**5ad**): FTIR (ATR, cm^−1^): 3472 (N-H), 1678 (C=O), 1142, 854, 748. ^1^H-NMR (300 MHz, DMSO-*d*_6_): δ = 1.28 (3H, t, *J =* 7.15 Hz, -CH_3_), 4.12 (2H, q, *J =* 7.15 Hz, -CH_2_), 4.89 (2H, s, -CH_2_-), 7.09 (1H, td, *J =* 9.30–2.40 Hz, Benzimidazole C-H), 7.28 (1H, td, *J =* 8.40 Hz - 2.40 Hz, Difluorophenyl C-H), 7.44–7.52 (3H, m, Difluorophenyl C-H, Benzimidazole C-H), 7.85 (2H, d, *J =* 8.40 Hz, Benzene C-H), 7.98–8.06 (1H, m, Difluorophenyl C-H), 8.32 (2H, d, *J =* 8.40 Hz, Benzene C-H), 13.23 (1H, s, Benzimidazole N-H). ^13^C-NMR (75 MHz, DMSO-*d*_6_): δ = 13.96, 15.48, 44.30, 99.28 (d, *J =* 27.8 Hz), 104.10 (d, *J =* 22.4 Hz), 105.79 (t, *J =* 26.6 Hz), 111.08 (d, *J =* 10.2 Hz), 113.02 (dd, *J =* 21.8 Hz， 3.2 Hz), 126.86, 127.39 (d, *J =* 9.1 Hz), 129.32, 131.61, 133.35 (dd, *J =* 10.8 Hz, 3.7 Hz), 134.42, 137.16, 150.47 (d, *J =* 12.2 Hz), , 151.65, 152.16 (d, *J =* 2.8 Hz), 154.77, 157.99 (d, *J =* 232.6 Hz), 159.28 (dd, *J =* 234.9 Hz, 12.3 Hz), 165.86 (dd, *J =* 254.1 Hz, 13.0 Hz), 190.26 (d, *J =* 4.2 Hz). HRMS (*m*/*z*): [M + H]+ calcd. for C_25_H_18_N_5_F_3_OS: 494.1257; found: 494.1242.

### 3.2. Antimicrobial Assay

With the use of fluconazole and ketoconazole as positive control drugs, microbiological studies were performed according to following guide: EUCAST definitive (EDef 7.1) method [67] for *C. albicans* (ATCC 24433), *C. krusei* (ATCC 6258), *C. parapsilosis* (ATCC 22019) and *C. glabrata* (ATCC 90030). MIC readings were carried out twice for each chemical agent. The yeasts were maintained in RPMI after overnight incubation at 37 °C. The inocula of test microorganisms adjusted to match the turbidity of a MacFarland 0.5 standard tube as determined with a spectrophotometer and the final inoculum size was 0.5–2.5 × 10^5^ cfu/mL for antifungal assay. Testing was carried out in RPMI at pH = 7 and the two-fold serial dilutions technique was applied. The last well on the microplates containing only inoculated broth was kept as controls and the last well with no growth of microorganism was recorded to represent the MIC_50_ expressed in μg/mL. For the antifungal assays, the compounds were dissolved in DMSO. Further dilutions of the compounds and standard drugs in test medium were prepared at the required quantities of 800, 400, 200, 100, 50, 25, 12.5, 6.25, 3.125, 1.5625 and 0.78 μg/mL concentrations RPMI. The completed plates were incubated for 24 h. At the end of the incubation, resazurin (20 µg/mL) was added into each well to control whether the growth in wells. Then, plates were allowed to incubate for 2 h. MIC_50_ values were determined using a microplate reader at 590 nm excitation, 560 nm emission. Each experiment in the antimicrobial assays was replicated twice in order to define the MIC_50_ values given in Table 2.

### 3.3. Prediction of ADME Proporties

In order to predict pharmacokinetic profiles of the target compounds (**5a**–**5ad**), some physicochemical parameters were calculated and ADME properties of the compounds were evaluated using the Molinspiration property calculation program [59].

### 3.4. Cytotoxicity Assay

Cytotoxicity was tested using the NIH/3T3 mouse embryonic fibroblast cell line (ATCC^®^ CRL-1658™, London, UK). NIH/3T3 cells were incubated according to the supplier’s recommendations and they were seeded at 1 × 10^4^ cells into each well of 96-well plates. MTT assay was performed as previously described [68,69]. The compounds were tested in a concentration range of 800 µg/mL and 0.78 µg/mL concentrations. Percent inhibition was calculated for each concentration according to the formula below, and IC_50_ values were determined by plotting a concentration–response curve of inhibition percent versus compound concentrations tested [70].

### 3.5. Quantification of Ergosterol Level

Total intracellular sterols were extracted as reported by Breivik and Owades [64] with slight modifications. Briefly, a single *C. albicans* colony from an overnight Sabouraud dextrose agar plate culture was used to inoculate 50 mL of Sabouraud dextrose broth (*Difco*) containing 0, 0.78, 1.56, and 3.12 µg/mL of compounds **5w**, **5ad**, fluconazole, and ketoconazole. The cultures were incubated for 16 h with shaking at 35 °C. The stationary-phase cells were harvested by centrifugation at 2700 rpm (Hettich, Rotina 380 R, Tuttlingen, Germany) for 5 min and washed once with sterile distilled water. Three milliliters of 25% alcoholic potassium hydroxide solution was added to each pellet and vortex mixed for 1 min. Cell suspensions were transferred to sterile borosilicate glass screw-cap tubes and were incubated in an 85 °C water bath for 1 h. Following incubation, tubes were allowed to cool to room temperature. Sterols were then extracted by addition of a mixture of 1 mL of sterile distilled water and 3 mL of chloroform followed by vigorous vortex mixing for 3 min. The chloroform layer was transferred to a clean borosilicate glass screw-cap tube and 1 µL of sterol extract was injected to LCMSMS system (Shimadzu LCMS 8040, Kyoto, Japan). The mass spectrometric analysis were achieved by employing the Nexera XR UFLC system coupled to an LCMS-8040 tandem quadrupole mass spectrometer (Shimadzu, Kyoto, Japan). Labsolutions LCMS software (Version 5.86, Shimadzu) was used to control the instruments and process the data. The Nexera UFLC system used in the analysis consisted of two pumps (LC-20ADxr), an autosampler (SIL-20ADxr), a column heater (CTO-10ASvp), and a degasser (DGU-20A5R). This instrument was equipped with ESI sources. Chromatographic separation was performed using a Shimadzu Shimpack FC-ODS C18 column (150 mm × 2.0 mm, 3 µm) at a flow rate of 0.25 mL/min in ESI source. The isocratic mobile phase consisted of acetonitrile-water 0.1% formic acid (50:50, *v*/*v*). The mass spectrometer operating parameters were optimized as follows: nebulizer gas flow, 3 L/min; drying gas flow, 15 L/min; desolvation line (DL) temperature, 250 °C; heat block temperature, 400 °C in ESI source. Other parameters were tuned automatically. MRM method was optimized using ergosterol standard stock solution with concentration of 20 µg/mL. Ergosterol quantity in negative control samples was regarded as 100%. All concentrations were analyzed in quadruplicate, and the results were expressed as mean ± standard deviation (SD).

### 3.6. Fluorescence Microscopy

The Live/Dead BacLight viability kit from Molecular Probes, Inc. (Eugene, OR, USA) was used as reported by Nobman et al. [65]. In this assay, the SYTO-9 and PI stains are in competition to bind to fungal nucleic acid. PI enters only cells with damaged membranes, while SYTO-9 marks cells with both damaged and unaffected membranes. A culture of *C. albicans* was grownup to mid-log phase in 20 mL of SDB. Twenty milliliters of the yeast culture was divided into two equal parts. Initial culture were incubated for 2 h with 3.12 µg/mL (4 × MIC_50_) of compound **5w**. The second culture was incubated under same conditions without a compound. Both cultures were centrifuged at 3000 rpm for 10 min. and then cell pellets were incubated with PI and SYTO9 at 40 °C for 30 min. The stained yeast cultures were observed under a fluorescence microscope (Carl-Zeis, Axio Scope.A1, Göttingen, Germany) using 100× oil-immersion objective [71].

## 4. Conclusions

In summary, preliminary evaluation of new 2-((5-(4-(5-substituted-*1H*-benzimidazol-2-yl)phenyl)-4-substituted-4*H*-1,2,4-triazol-3-yl)thio)-1-(substitutedphenyl)ethan-1-one derivatives as antifungal agents result in promising findings. Compounds **5w** and **5ad** exerted a good antifungal profile. Furthermore, toxicological and ADME studies indicated the relative potency of compounds **5w** and **5ad**. Results of ergosterol level quantification assay and fluorescence microscopy studies revealed that the mechanism of action of compounds is associated with the inhibition of ergosterol biosynthesis which may subsequently results in altered membrane fluidity, plasma membrane biogenesis and functions of fungi. Consequently, all these data may pave the way for researchers to synthesize similar compounds possessing enhanced antimicrobial profile.

## Figures and Tables

**Table 1:** Some characteristics of compounds (**5a**–**5ad**).

Compound	R1	R2	R3	R4	m.p. (°C)	Yield (%)
**5a**	-H	-CH_3_	-H	-H	269.5	81
**5b**	-H	-CH_3_	-H	-Cl	280.6	82
**5c**	-H	-CH_3_	-H	-F	259.8	80
**5d**	-H	-CH_3_	-Cl	-Cl	234.7	76
**5e**	-H	-CH_3_	-F	-F	256.2	75
**5f**	-H	-C_2_H_5_	-H	-H	220.9	78
**5g**	-H	-C_2_H_5_	-H	-Cl	242.6	81
**5h**	-H	-C_2_H_5_	-H	-F	231.3	80
**5i**	-H	-C_2_H_5_	-Cl	-Cl	221.7	83
**5j**	-H	-C_2_H_5_	-F	-F	238.2	86
**5k**	-Cl	-CH_3_	-H	-H	260.0	87
**5l**	-Cl	-CH_3_	-H	-Cl	236.9	77
**5m**	-Cl	-CH_3_	-H	-F	274.5	82
**5n**	-Cl	-CH_3_	-Cl	-Cl	274.2	77
**5o**	-Cl	-CH_3_	-F	-F	264.9	85
**5p**	-Cl	-C_2_H_5_	-H	-H	248.5	80
**5q**	-Cl	-C_2_H_5_	-H	-Cl	252.2	91
**5r**	-Cl	-C_2_H_5_	-H	-F	265.5	78
**5s**	-Cl	-C_2_H_5_	-Cl	-Cl	192.0	77
**5t**	-Cl	-C_2_H_5_	-F	-F	250.6	74
**5u**	-F	-CH_3_	-H	-H	277.1	81
**5v**	-F	-CH_3_	-H	-Cl	252.9	84
**5w**	-F	-CH_3_	-H	-F	227.4	82
**5x**	-F	-CH_3_	-Cl	-Cl	228.5	89
**5y**	-F	-CH_3_	-F	-F	263.2	88
**5z**	-F	-C_2_H_5_	-H	-H	253.6	90
**5aa**	-F	-C_2_H_5_	-H	-Cl	241.4	79
**5ab**	-F	-C_2_H_5_	-H	-F	251.0	81
**5ac**	-F	-C_2_H_5_	-Cl	-Cl	223.3	87
**5ad**	-F	-C_2_H_5_	-F	-F	234.2	76

**Table 2:** MIC_50_ (µg/mL) values of compounds (**5a**–**5ad**).

Compound	*C. albicans*	*C. glabrata*	*C. krusei*	*C. parapsilopsis*
**5a**	100	100	100	100
**5b**	25	50	50	50
**5c**	12.5	25	25	12.5
**5d**	25	50	50	25
**5e**	12.5	25	25	25
**5f**	100	100	100	100
**5g**	25	50	25	50
**5h**	12.5	25	12.5	25
**5i**	25	50	50	50
**5j**	12.5	25	12.5	25
**5k**	12.5	25	25	12.5
**5l**	6.25	12.5	6.25	12.5
**5m**	0.78	1.56	0.78	0.78
**5n**	1.56	3.12	1.56	3.12
**5o**	0.78	1.56	1.56	0.78
**5p**	12.5	12.5	50	25
**5q**	6.25	12.5	12.5	12.5
**5r**	0.78	0.78	1.56	1.56
**5s**	1.56	3.12	3.12	1.56
**5t**	0.78	1.56	0.78	0.78
**5u**	12.5	25	50	50
**5v**	12.5	6.25	12.5	12.5
**5w**	0.78	0.78	0.78	0.78
**5x**	3.12	1.56	3.12	3.12
**5y**	0.78	1.56	0.78	1.56
**5z**	12.5	25	50	50
**5aa**	6.25	12.5	6.25	12.5
**5ab**	0.78	1.56	1.56	0.78
**5ac**	1.56	1.56	1.56	1.56
**5ad**	0.78	0.78	1.56	0.78
Ketoconazole	0.78	1.56	1.56	1.56
Fluconazole	0.78	1.56	1.56	0.78

**Table 3:** Calculated ADME parameters and DLS of compounds **5a**–**5ad**.

Compound	MW	logP	tPSA	nON	nOHNH	MV	Vio
**5m**	477.95	6.07	76.47	6	1	389.09	1
**5n**	528.85	7.19	76.47	6	1	411.23	2
**5o**	495.94	6.16	76.47	6	1	394.02	1
**5r**	491.98	6.44	76.47	6	1	405.89	1
**5s**	542.88	7.56	76.47	6	1	428.03	2
**5t**	509.97	6.53	76.47	6	1	410.82	2
**5w**	461.50	5.55	76.47	6	1	380.48	1
**5x**	512.40	6.67	76.47	6	1	402.62	2
**5y**	479.49	5.64	76.47	6	1	385.41	1
**5ab**	475.52	5.93	76.47	6	1	397.28	1
**5ac**	526.42	7.05	76.47	6	1	419.42	2
**5ad**	493.51	6.02	76.47	6	1	402.21	1
Ketoconazole	531.44	3.77	69.08	8	0	452.47	1
Fluconazole	320.30	0.05	81.66	7	1	266.11	0

MW: Molecular weight; log P: log octanol/water partition coefficient; tPSA: Total Polar Surface Area; nON: number of Hydrogen acceptors; nOHNH: number of Hydrogen donors and MV: Molecular Volume were calculated using Molinspiration Calculation of Molecular Properties toolkit. Vio: Violation number Lipinski’s rule.

**Table 4:** IC_50_ of the selected compounds on NIH/3T3 mouse embryonic fibroblast cell lines (ATCC CRL1658).

Compound	IC_50_ (µg/mL)
**5m**	17.38 ± 0.89
**5o**	1.63 ± 0.02
**5r**	13.94 ± 0.69
**5w**	65.28 ± 2.94
**5y**	11.14 ± 0.57
**5ab**	43.13 ± 2.88
**5ad**	119.55 ± 4.39

## Supplementary Materials

Supplementary materials are available online. The spectra of compounds **5w** and **5ad** are available online.

## References

[1] Whibley N., Gaffen S.L. (2015). Beyond *Candida* albicans: Mechanisms of immunity to non-albicans *Candida* species. Cytokine.

[2] Sparber F., LeibundGut-Landmann S. (2015). Interleukin 17-mediated host defense against *Candida albicans*. Pathogens.

[3] Jiang Y., Cao Y., Zhang J., Zou Y., Chai X., Hu H., Zhao Q., Wu Q., Zhang D., Jiang Y. (2011). Design, synthesis and antifungal evaluation of 1-(2-(2,4-difluorophenyl)-2-hydroxy-3-(1*H*-1,2,4-triazol-1-yl)propyl)-1*H*-1,2,4-triazol-5(4*H*)-one. Eur. J. Med. Chem..

[4] Altıntop M.D., Kaplancıklı Z.A., Turan-Zitouni G., Özdemir A., İşcan G., Akalın G., Yıldırım Ş. (2011). Synthesis and anticandidal activity of new triazolothiadiazine derivatives. Eur. J. Med. Chem..

[5] Georgopapadakou N.H., Walsh T.J. (1994). Human mycoses: Drugs and targets for emerging pathogens. Science.

[6] Silvestri R., Artico M., La Regina G., Di Pasquali A., De Martino G., D′Auria F.D., Nencioni L., Palamara A.T. (2004). Imidazole analogues of fluoxetine, a novel class of anti-*Candida* agents. J. Med. Chem..

[7] DiDomenico B. (1999). Novel antifungal drugs. Curr. Opin. Microbiol..

[8] Beck–Sague C., Jarvis W. (1993). Secular trends in the epidemiology of nosocomial fungal infections in the United States, 1980–1990. J. Infect. Dis..

[9] Georgopapadakou N.H. (1998). Antifungals: Mechanism of action and resistance, established and novel drugs. Curr. Opin. Microbiol..

[10] Wang W., Wang S., Liu Y., Dong G., Cao Y., Miao Z., Yao J., Zhang W., Sheng C. (2010). Novel conformationally restricted triazole derivatives with potent antifungal activity. Eur. J. Med. Chem..

[11] Jiang Z., Wang Y., Wang W., Wang S., Xu B., Fan G., Dong G., Liu Y., Yao J., Miao Z., Zhang W., Sheng C. (2013). Discovery of highly potent triazole antifungal derivatives by heterocycle-benzene bioisosteric replacement. Eur. J. Med. Chem..

[12] Stefanska J., Szulczyk D., Koziol A.E., Miroslaw B., Kedzierska E., Fidecka S., Busonera B., Sanna G., Giliberti G., La Colla P. (2012). Disubstituted thiourea derivatives and their activity on CNS: Synthesis and biological evaluation. Eur. J. Med. Chem..

[13] Muralikrishna A., Venkatesh B.C., Padmavathi V., Padmaja A., Kondaiah P., Krishna N.S. (2012). Synthesis, antimicrobial and cytotoxic activities of sulfone linked bisheterocycles. Eur. J. Med. Chem..

[14] Zoumpoulakis P., Camoutsis C.H., Pairas G., Soković M., Glamočlija J., Potamitis C., Pitsas A. (2012). Synthesis of novel sulfonamide-1,2,4-triazoles, 1,3,4-thiadiazoles and 1,3,4-oxadiazoles, as potential antibacterial and antifungal agents, biological evaluation and conformational analysis studies. Bioorg. Med. Chem..

[15] Kathiravan M.K., Salake A.B., Chothe A.S., Dudhe P.B., Watode R.P., Mukta M.S., Gadhwe S. (2012). The biology and chemistry of antifungal agents: a review. Bioorg. Med. Chem..

[16] Spasov A.A., Yozhitsa I.N., Bugaeva L.I., Anisimova V.A. (1999). Benzimidazole derivatives: Spectrum of pharmacological activity and toxicological properties (a review). Pharm. Chem. J..

[17] Arjmand F., Mohani B., Ahmad S. (2005). Synthesis, antibacterial, antifungal activity and interaction of CT-DNA with a new benzimidazole derived Cu(II) complex. Eur. J. Med. Chem..

[18] Shaker Y.M., Omar M.A., Mahmoud K., Elhallouty S.M., El-Senousy W.M., Ali M.M., Mahmoud A.E., Abdel-Halim A.H., Soliman S.M., El-Divani H.I. (2015). Synthesis, in vitro and in vivo antitumor and antiviral activity of novel 1-substituted benzimidazole derivatives. J. Enzyme Inhib. Med. Chem..

[19] Tonelli M., Boido V., La Colla P., Loddo R., Posocco P., Paneni M.S., Fermeglia M., Pricl S. (2010). Pharmacophore modeling, resistant mutant isolation, docking, and MM-PBSA analysis: Combined experimental/computer-assisted approaches to identify new inhibitors of the bovine viral diarrhea virus (BVDV). Bioorg. Med. Chem..

[20] Desai N.C., Kotadiya G.M. (2014). Microwave-assisted synthesis of benzimidazole bearing 1,3,4-oxadiazole derivatives: Screening for their in vitro antimicrobial activity. Med. Chem. Res..

[21] Desai N.C., Shihory N., Bhatt M., Patel B., Karkar T. (2015). Studies on antimicrobial evaluation of some 1-((1-(1*H*-benzo[*d*]imidazol-2-yl) ethylidene) amino)-6-((arylidene)amino)-2-oxo-4-phenyl-1,2-dihydropyridine-3,5-dicarbonitriles. Synth. Commun..

[22] Desai N.C., Kotadiya G.M. (2014). Synthesis, antimicrobial, and cytotoxic activities of novel benzimidazole derivatives bearing cyanopyridine and 4-thiazolidinone motifs. Med. Chem. Res..

[23] Subhashini N.J.P., Boddu L., Amanaganti J. (2014). Synthesis, characterization, and antimicrobial activity of new bis-1,2,3-triazol-*H*-yl-substituted 2-arylbenzimidazoles. Russ. J. Gen. Chem..

[24] Montalvão S., Leino T.O., Kiuru P.S., Lillsunde K.E., Yli-Kauhaluoma J., Tammela P. (2016). Synthesis and biological evaluation of 2-aminobenzothiazole and benzimidazole analogs based on the clathrodin structure. Arch. Pharm. Chem. Life Sci..

[25] Joshi D., Parikh K. (2014). Synthesis and evaluation of novel benzimidazole derivatives as antimicrobial agents. Med. Chem. Res..

[26] Jiang L., Wang M.Y., Wan F.X., Qu Z.Q. (2015). Synthesis and biological activity of tri-substituted 1,2,4-triazoles bearing benzimidazole moiety. Phosphorus Sulfur Silicon Relat. Elem..

[27] Mavrova A.T., Yancheva D., Anastassova N., Anichina K., Zvezdanovic J., Djordjevic A., Markovic D., Smelcerovic A. (2015). Synthesis, electronic properties, antioxidant and antibacterial activity of some new benzimidazoles. Bioorg. Med. Chem..

[28] Keller P., Müller C., Engelhardt I., Hiller E., Lemuth K., Eickhoff H., Wiesmüller K.H., Burger-Kentischer A., Bracher F., Rupp S. (2015). An antifungal benzimidazole derivative inhibits ergosterol biosynthesis and reveals novel sterols. Antimicrob. Agents Chemother..

[29] Soodamani V., Patel D., Nayakanti D., Josyula R. (2015). An efficient synthesis of novel 2-(5-indolyl)-1*H*-benzimidazole derivatives and evaluation of their antimicrobial activities. J. Heterocycl. Chem..

[30] Jiang Y., Han Q., Shen R., Zang X., Wang B. (2014). Synthesis and antimicrobial activity of some new 4*H*-pyrrolo [1,2-*a*]benzimidazoles. Chem. Res. Chin. Univ..

[31] Desai N.C., Pandya D.D., Kotadiya M.G., Desai P. (2013). Synthesis of pyridyl benzimidazoles encompassing 4-thiazolidinone derivatives as potential antimicrobial agents. Lett. Drug Des. Discov..

[32] Vashist N., Sambi S.S., Kumar P., Narasimhan B. (2015). Development of QSAR for antimicrobial activity of substituted benzimidazoles. Drug Res..

[33] Tonelli M., Novelli F., Tasso B., Vazzana I., Sparatore A., La Colla P., Sanna G., Giliberti G., Busonera B., Farci P. (2014). Antiviral activity of benzimidazole derivatives. III. Novel anti-CVB-5, anti-RSV and anti-Sb-1 agents. Bioorg. Med. Chem..

[34] Kaplancikli Z.A., Turan-Zitouni G., Revial G., Guven K. (2004). Synthesis and study of antibacterial and antifungal activities of novel 2-[[(benzoxazole/benzimidazole-2-yl) sulfanyl] acetylamino] thiazoles. Arch. Pharm. Res..

[35] Özdemir A., Turan-Zitouni G., Asım Kaplancıklı Z., Revial G., Demirci F., İşcan G. (2010). Preparation of some pyrazoline derivatives and evaluation of their antifungal activities. J. Enzyme Inhib. Med. Chem..

[36] Altintop M.D., Mohsen U.A., Ozkay Y., Demirel R., Kaplancikli ZA. (2015). Synthesis and antimicrobial activity of benzimidazole-based acetamide derivatives. Turk. J. Pharm. Sci..

[37] Yurttas L., Ozkay Y., Karaca H., Tunali Y., Kaplancıkli ZA. (2013). Synthesis and antimicrobial evaluation of some 2,5-disubstituted benzimidazole derivatives. Lett. Drug Des. Discov..

[38] Ozkay Y., Tunalı Y., Karaca H., Işıkdağ İ. (2011). Antimicrobial activity of a new series of benzimidazole derivatives. Arc. Pharm. Res..

[39] Ozkay Y., Tunalı Y., Karaca H., Işıkdağ İ. (2011). Antimicrobial activity of a new combination system of benzimidazole and various azoles. Arc. Pharm..

[40] Ozkay Y., Tunalı Y., Karaca H., Işıkdağ İ. (2010). Antimicrobial activity and a SAR study of some novel benzimidazole derivatives bearing hydrazone moiety. Eur. J. Med. Chem..

[41] Ozkay Y., Tunali Y., Karaca H., Isikdag I. (2011). Synthesis and antimicrobial activity of some novel benzimidazole hydrazides. Asian J. Chem..

[42] Acar-Çevik U., Sağlık B.N., Ozkay Y., Canturk Z., Bueno J., Demirci F., Koparal A.S. (2017). Synthesis of new fluoro-benzimidazole derivatives as an approach towards the discovery of novel Intestinal antiseptic drug candidates. Curr. Pharm. Des..

[43] Yadagiri B., Lown J.W. (1990). Convenient routes to substituted benzimidazoles and imidazolo[4,5-*b*]pyridines using nitrobenzene as oxidant. Synth. Commun..

[44] Verner E., Katz B.A., Spencer J.R., Allen D., Hataye J., Hruzewicz W., Hui H.C., Kolesnikov A., Li Y., Luong C. (2001). Development of serine protease inhibitors displaying a multicentered short (<2.3 Å) hydrogen bond binding mode:  Inhibitors of urokinase-type plasminogen activator and factor Xa. J. Med. Chem..

[45] Bhatnagar I., George M.V. (1968). Oxidation with metal oxides-II: Oxidation of chalcone phenylhydrazones, pyrazolines, *o*-aminobenzylidine anils and o-hydroxy benzylidine anils with manganese dioxide. Tetrahedron.

[46] Patzold F., Zeuner F., Heyer T.H., Niclas H. (1992). Dehydrogenations using benzofuroxan as oxidant. J. Synth. Commun..

[47] Chikashita H., Nishida S., Miyasaki M., Morita Y., Itoh K. (1987). Insitu generation and synthetic application of 2-phenylbenzimidazoline to the selective reduction of carbon carbon double-bonds of electron-deficient olefins. Bull. Chem. Soc. Jpn..

[48] Beaulieu P.L., Hache B., Von Moos E. (2003). A practical Oxone (*R*)-mediated, high-throughput, solution-phase synthesis of benzimidazoles from 1,2-phenylenediamines and aldehydes and its application to preparative scale synthesis. Synthesis.

[49] Stephens F.F., Bower J.D. (1949). The preparation of benziminazoles and benzoxazoles from Schiff′s bases. Part I. J. Chem. Soc..

[50] Ridley H.F., Spickett R.G.W., Timmis G.M. (1965). A new synthesis of benzimidazoles and aza-analogs. J. Heterocycl. Chem..

[51] Algul O., Kaessler A., Apcin Y., Yilmaz A., Jose J. (2008). Comparative studies on conventional and microwave synthesis of some benzimidazole, benzothiazole and indole derivatives and testing on inhibition of hyaluronidase. Molecules.

[52] Yamashita T., Yamada S., Yamazaki Y., Tanaka H. (2009). New procedure for the synthesis of 2-alkylbenzimidazoles. Synth. Commun..

[53] Navarrete-Vázquez G., Moreno-Diaz H., Aguirre-Crespo F., León-Rivera I., Villalobos-Molina R., Muñoz-Muñiz O., Estrada-Soto S. (2007). Design, microwave-assisted synthesis, and spasmolytic activity of 2-(alkyloxyaryl)-1*H*-benzimidazole derivatives as constrained stilbene bioisosteres. Bioorg. Med. Chem. Lett..

[54] Navarrete-Vázquez G., Moreno-Diaz H., Estrada-Soto S., Torres-Piedra M., León-Rivera I., Tlahuext H., Muñoz-Muñiz O., Torres-Gómez H. (2007). Microwave assisted one pot synthesis of 2-(substitutedphenyl)-1*H*-benzimidazole derivatives. Synth. Commun..

[55] El kihel A., Essassi E.M., Bauchat P. (2012). ^1^H- and ^13^C-NMR spectra of condensed benzimidazole and imidazobenzodiazepines. Arab. J. Chem..

[56] Borra R.C., Lotufo M.A., Gagioti S.M., Barros F.D.M., Andrade PM. (2009). A simple method to measure cell viability in proliferation and cytotoxicity assays. Braz. Oral Res..

[57] Palomino J.C., Martin A., Camacho M., Guerra H., Swings J., Portaels F. (2002). Resazurin microtiter assay plate: Simple and inexpensive method for detection of drug resistance in Mycobacterium tuberculosis. Antimicrob. Agents Chemother..

[58] Van De Waterbeemd H., Gifford E. (2003). ADMET in silico modelling: Towards prediction paradise?. Nat. Rev. Drug Discov..

[59] Molinspiration Cheminformatics, Bratislava, Slovak Republic. http://www.molinspiration.com/services/properties.html.

[60] Lipinski C.A., Lombardo F., Dominy B.W., Feeney P.J. (2001). Experimental and computational approaches to estimate solubility and permeability in drug discovery and development settings. Adv. Drug Deliv. Rev..

[61] Kramer J.A., Sagartz J.E., Morris D.L. (2007). The application of discovery toxicology and pathology towards the design of safer pharmaceutical lead candidates. Nat. Rev. Drug Discov..

[62] International Organization for Standardization (2009). Biological Evaluation of Medical Devices-Part 5: Tests for In Vitro Cytotoxicity ISO-10993-5.

[63] Lupetti A., Danesi R., Campa M., Del Tacca M., Kelly S. (2002). Molecular basis of resistance to azole antifungals. Trends Mol. Med..

[64] Breivik O.N., Owades J.L. (1957). Spectrophotometric semi-microdetermination of ergosterol in yeast. Agric. Food Chem..

[65] Zhang Y.Q., Rao R. (2010). Beyond ergosterol: Linking pH to antifungal mechanisms. Virulence.

[66] Nobmann P., Bourke P., Dunne J., Henehan G. (2010). In vitro antimicrobial activity and mechanism of action of novel carbohydrate fatty acid derivatives against Staphylococcus aureus and MRSA. J. Appl. Microbiol..

[67] EUCAST (2008). Definitive Document EDef 7.1: Method for the Determination of Broth Dilution MICs of Antifungal Agents for Fermentative Yeasts. Clin. Microbiol. Infect..

[68] Sağlık B.N., Ilgın S., Özkay Y. (2016). Synthesis of new donepezil analogues and investigation of their effects on cholinesterase enzymes. Eur. J. Med. Chem..

[69] Özkay Ü.D., Can Ö.D., Sağlık B.N., Çevik U.A., Levent S., Özkay Y., Atlı Ö. (2016). Design, synthesis, and AChE inhibitory activity of new benzothiazole–piperazines. Bioorg. Med. Chem. Lett..

[70] Patel S., Gheewala N., Suthar A., Shah A. (2009). In-vitro cytotoxicity activity of Solanum nigrum extract against Hela cell line and Vero cell line. Int. J. Pharm. Pharm. Sci..

[71] Li Y., Smith C., Wu H., Padhee S., Manoj N., Joseph C., Qiao Q., Chuanhai C., Yin H., Cai J. (2014). Lipidated Cyclic AApeptides Display Both Antimicrobial and Antiinflammatory Activity. ACS Chem. Biol..

