# Supplementary material for: New Benzimidazole-1,2,4-Triazole Hybrid Compounds: Synthesis, Anticandidal Activity and Cytotoxicity Evaluation
Source: Molecules. 2017 Mar 27;22(4):507. doi: 10.3390/molecules22040507 (PMC6154534; doi:10.3390/molecules22040507)
Supplement: Supplementary file 1 [file molecules-22-00507-s001.pdf]

New Benzimidazole-1,2,4-Triazole Hybrid Compounds: Synthesis, Anticandidal Activity and Cytotoxicity Evaluation

Hülya Karaca Gençer , Ulviye Acar Çevik, Serkan Levent, Begüm Nurpelin Sağlık, Büşra Korkut, Yusuf Özkay, Sinem Ilgın, Yusuf Öztürk

Spectra for the most active compounds 5w and 5ad.

DOPNALAB

	Item	Value
	Acquired Date&Time	26.01.2017 11:24:21
	Acquired by	System Administrator
	Filename	C:\Users\dopnalab\Desktop\Serkan\tu serisi\tu-431.ispd
	Spectrum name	tu-431
	Sample name	tu-43
	Sample ID	
	Option	
	Comment	
	No. of Scans	10
	Resolution	4 [cm-1]
	Apodization	Happ-Genzel

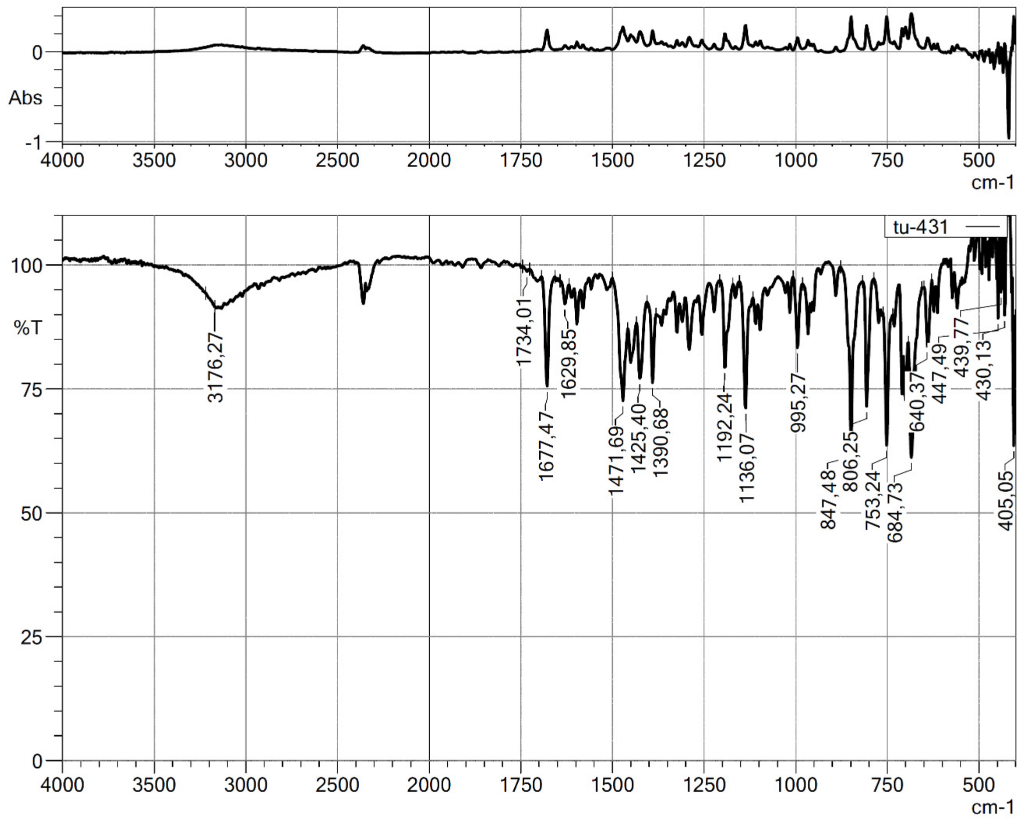

Figure SI-1. IR spectra of compound **5w**.

Formula Predictor Report - TU-43_61.lcd

Data File: C:\ LabSolutions\ Data\Analiz\uac\ TU-43_61.lcd

Elmt	Val.	Min	Max	Elmt	Val.	Min	Max	Elmt	Val.	Min	Max	Use Adduct
H	1	17	19	O	2	1	3	Cl	1	0	3	H
C	4	22	40	F	1	0	2	Br	1	0	1	
N	3	5	6	S	2	1	1					

Error Margin (ppm):	5	DBE Range:	17.0 - 30.0	Electron Ions:	both
HC Ratio:	unlimited	Apply N Rule:	yes	Use MSn Info:	no
Max Isotopes:	3	Isotope RI (%):	1.00	Isotope Res:	10000
MSn Iso RI (%):	10.00	MSn Logic Mode:	AND	Max Results:	500

Event#: 1 MS(E+) Ret. Time : 6.747 -> 6.747 Scan# : 1013 -> 1013

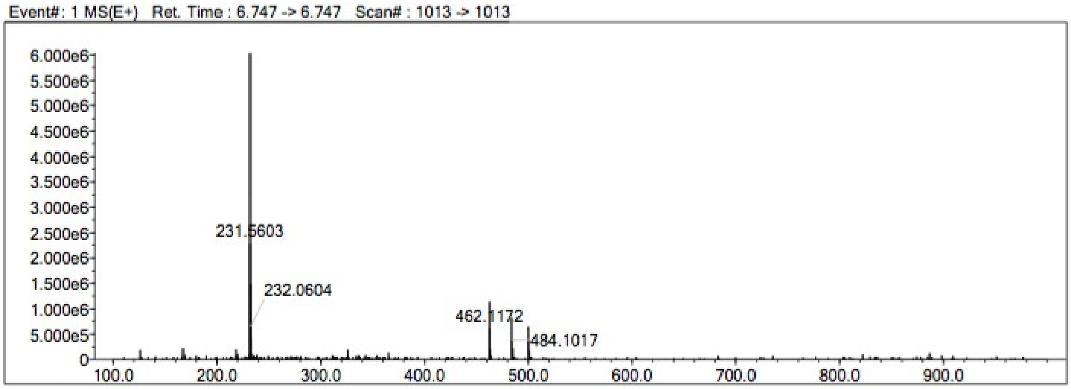

Measured region for 462.1172 m/ z

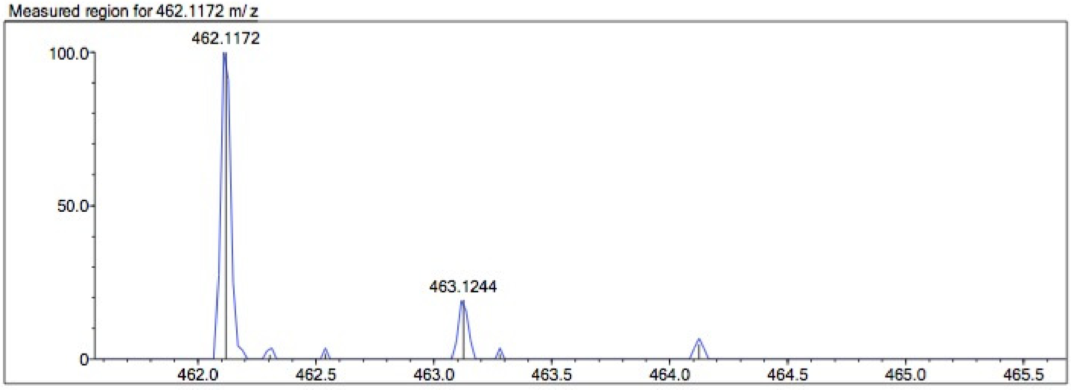

C24 H17 N5 O F2 S [M+H]+ : Predicted region for 462.1195 m/z

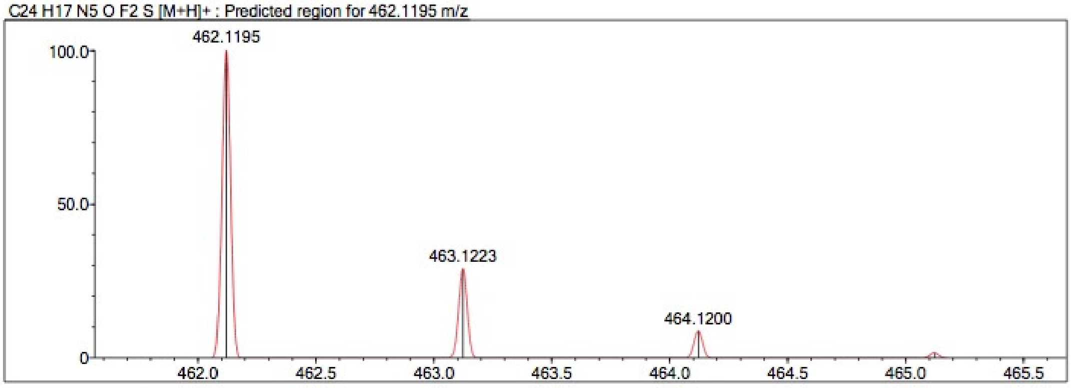

Rank	Score	Formula (M)	Ion	Meas. m/ z	Pred. m/ z	Df. (mDa)	Df. (ppm)	Iso	DBE
1	58.68	C24 H17 N5 O F2 S	[M+H]+	462.1172	462.1195	-2.3	-4.98	65.17	18.0

Figure SI-2. HRMS spectra of compound **5w**.

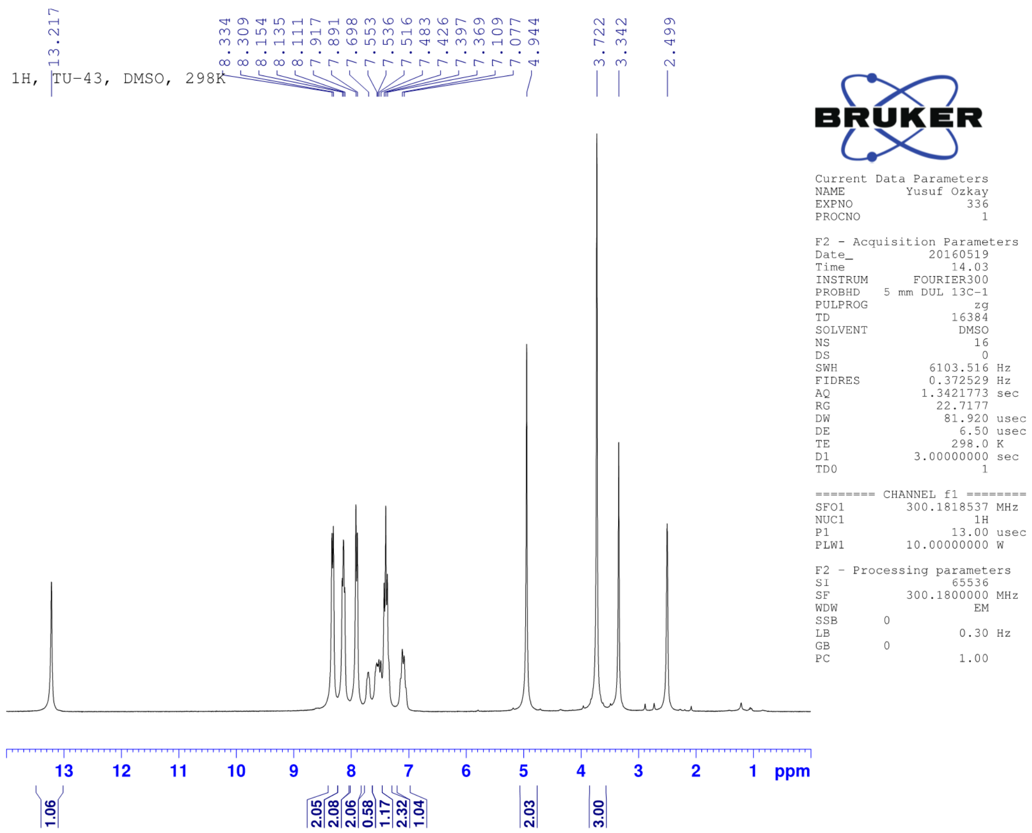

Figure SI-3. ^{1}H-NMR spectrum of compound **5w**.

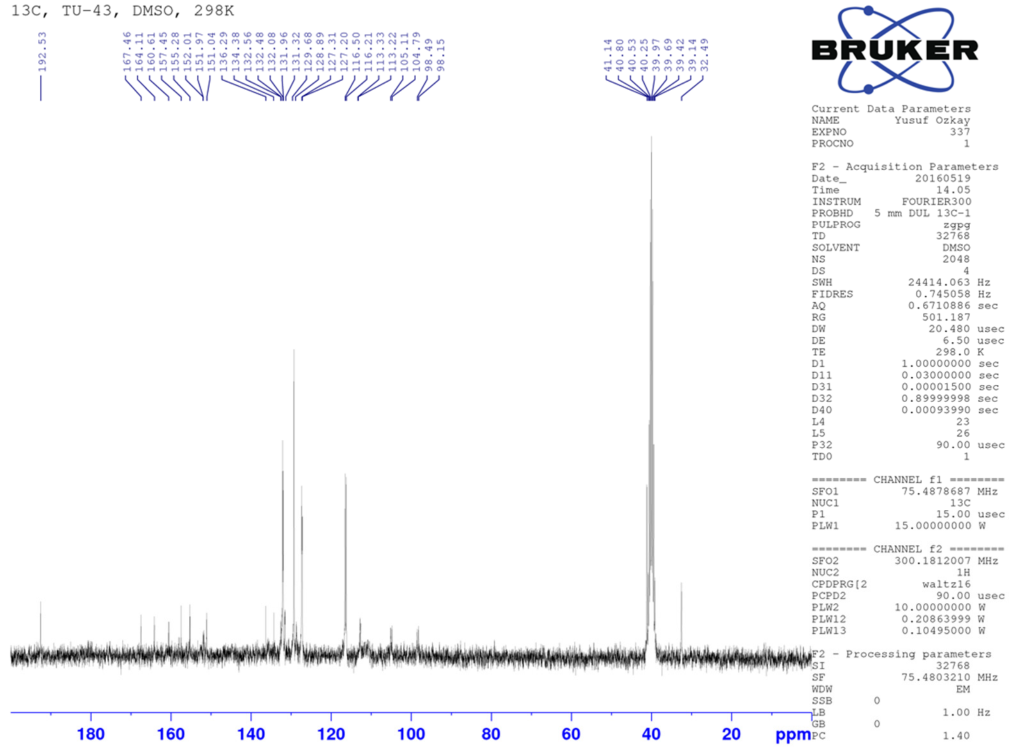

Figure SI-4. ^{13}C-NMR spectra of compound **5w**.

DOPNALAB

	Item	Value
	Acquired Date&Time	26.01.2017 11:45:58
	Acquired by	System Administrator
	Filename	C:\Users\dopnalab\Desktop\Serkan\tu serisi\tu-561.ispd
	Spectrum name	tu-561
	Sample name	tu-56
	Sample ID	
	Option	
	Comment	
	No. of Scans	10
	Resolution	4 [cm-1]
	Apodization	Happ-Genzel

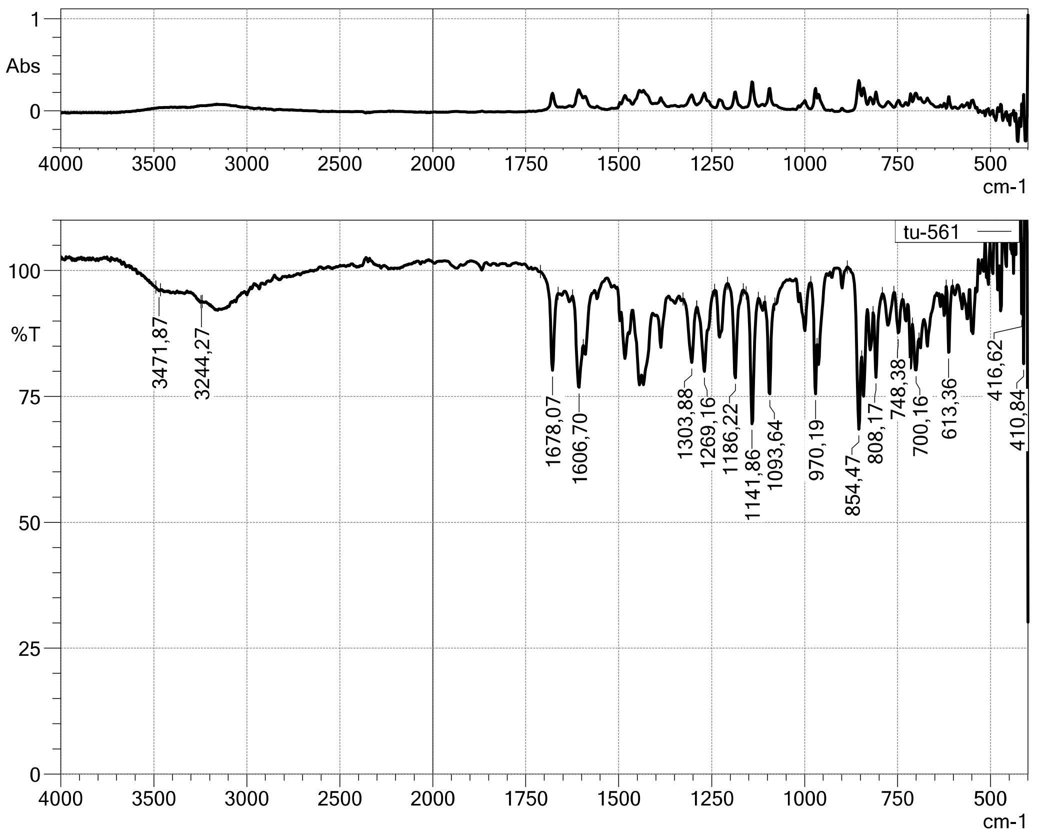

Figure SI-5. IR spectra of compound **5ad**.

Data File: C:\ LabSolutions\ Data\Analiz\uac\ TU-56_77.lcd

Elmt	Val.	Min	Max	Elmt	Val.	Min	Max	Elmt	Val.	Min	Max	Use Adduct
H	1	15	25	O	2	1	3	Cl	1	0	3	H
C	4	22	40	F	1	0	3	Br	1	0	1	
N	3	5	6	S	2	1	1					

Error Margin (ppm):	5	DBE Range:	17.0 - 21.0	Electron Ions:	both
HC Ratio:	unlimited	Apply N Rule:	yes	Use MSn Info:	no
Max Isotopes:	3	Isotope RI (%):	1.00	Isotope Res:	10000
MSn Iso RI (%):	10.00	MSn Logic Mode:	AND	Max Results:	500

Event#: 1 MS(E+) Ret. Time : 6.880 -> 6.880 Scan# : 1033 -> 1033

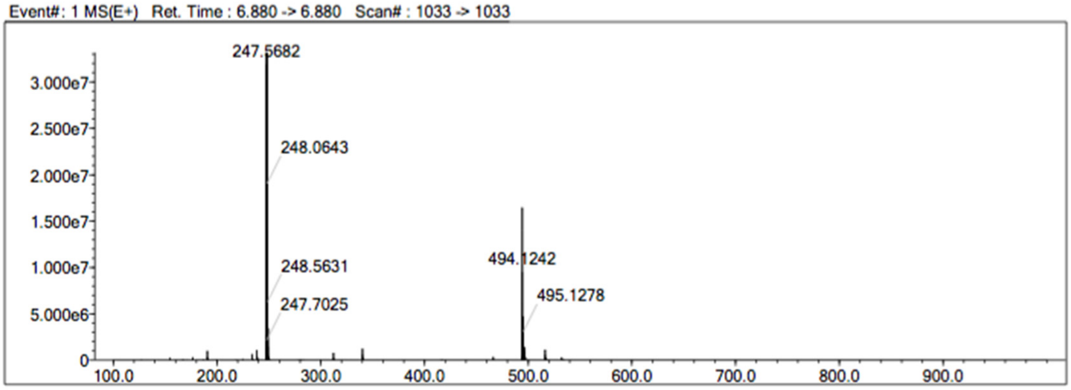

Measured region for 494.1242 m/z

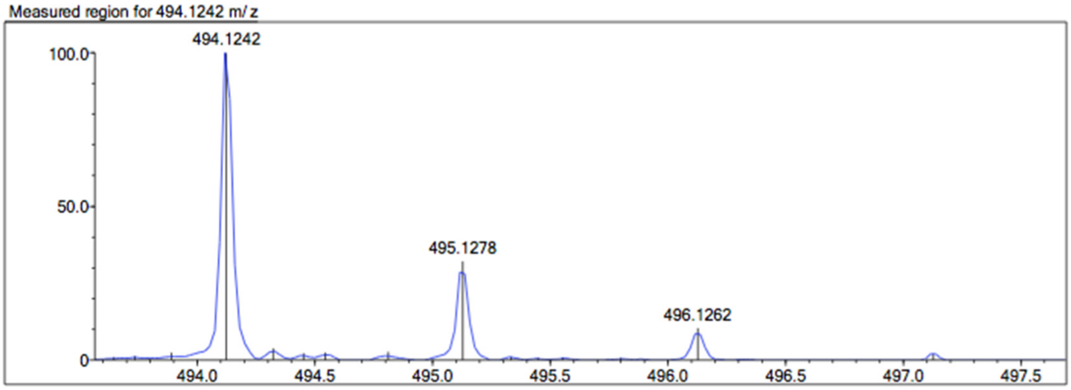

C25 H18 N5 O F3 S [M+H]+ : Predicted region for 494.1257 m/z

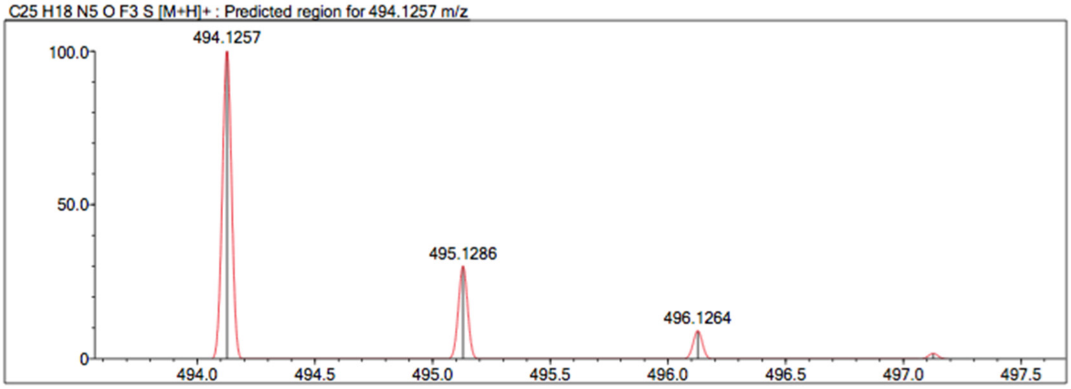

Rank	Score	Formula (M)	Ion	Meas. m/z	Pred. m/z	Df. (mDa)	Df. (ppm)	Iso	DBE
1	84.53	C25 H18 N5 O F3 S	[M+H]+	494.1242	494.1257	-1.5	-3.04	89.07	18.0

Figure SI-6. HRMS spectra of compound **5ad**.

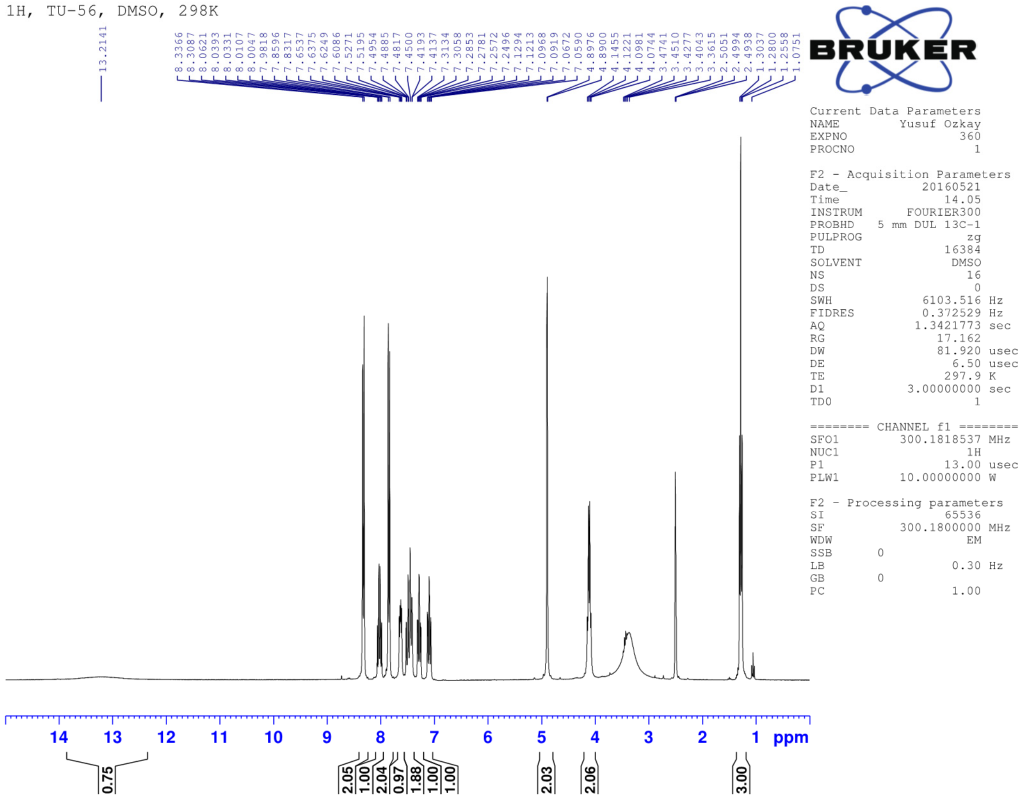

Figure SI-7. ^{1}H-NMR spectrum of compound **5ad**.

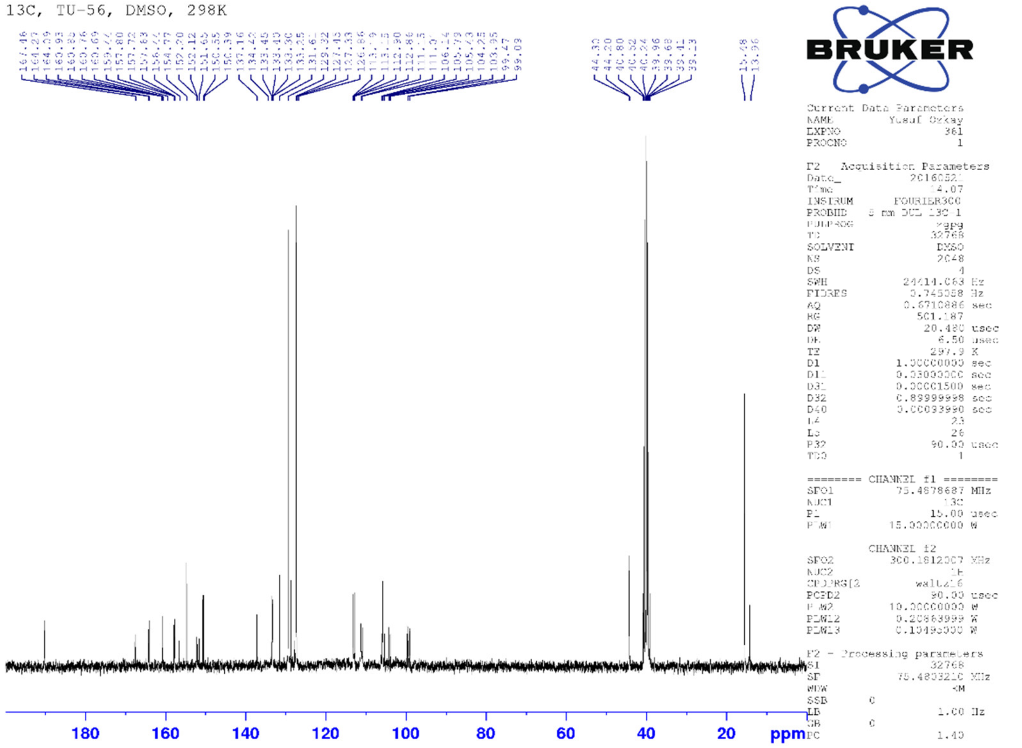

Figure SI-8. ^{13}C-NMR spectra of compound **5ad**.
